# Genotype, Age, Genetic Background, and Sex Influence *Epha2*-Related Cataract Development in Mice

**DOI:** 10.1167/iovs.62.12.3

**Published:** 2021-09-08

**Authors:** Alpana Dave, Jamie E. Craig, Mohammad Alamein, Karina Skrzypiec, Justin Beltz, Annalise Pfaff, Kathryn P. Burdon, Nuran Ercal, Robb U. de Iongh, Shiwani Sharma

**Affiliations:** 1 Department of Ophthalmology, Flinders University, Bedford Park, South Australia, Australia; 2 Department of Chemistry, Missouri University of Science and Technology, Rolla, Missouri, United States; 3 Menzies Institute for Medical Research, University of Tasmania, Hobart, Tasmania, Australia; 4 Ocular Development Laboratory, Anatomy & Neuroscience, University of Melbourne, Parkville, Australia

**Keywords:** *Epha2*, biological factors, mouse model, age-related cataract

## Abstract

**Purpose:**

Age-related cataract is the leading cause of blindness worldwide. Variants in the *EPHA2* gene increase the disease risk, and its knockout in mice causes cataract. We investigated whether age, sex, and genetic background, risk factors for age-related cataract, and *Epha2* genotype influence *Epha2*-related cataract development in mice.

**Methods:**

Cataract development was monitored in *Epha2^+/+^*, *Epha2^+/^^−^*, and *Epha2^−^^/^^−^* mice (*Epha2^Gt(KST085)Byg^*) on C57BL/6J and FVB:C57BL/6J (50:50) backgrounds. Cellular architecture of lenses, endoplasmic reticulum (ER) stress, and redox state were determined using histological, molecular, and analytical techniques.

**Results:**

*Epha2^−^^/^^−^* and *Epha2^+/^^−^* mice on C57BL/6J background developed severe cortical cataracts by 18 and 38 weeks of age, respectively, compared to development of similar cataract significantly later in *Epha2^−^^/^^−^* mice and no cataract in *Epha2^+/^^−^* mice in this strain on FVB background, which was previously reported. On FVB:C57BL/6J background, *Epha2^−^^/^^−^* mice developed severe cortical cataract by 38 weeks and *Epha2^+/^^−^* mice exhibited mild cortical cataract up to 64 weeks of age. Progression of cataract in *Epha2^−^^/^^−^* and *Epha2^+/^^−^* female mice on C57BL/6J and mixed background, respectively, was slower than in matched male mice. N-cadherin and β-catenin immunolabeling showed disorganized lens fiber cells and disruption of lens architecture in *Epha2^−^^/^^−^* and *Epha2^+/^^−^* lenses, coinciding with development of severe cataracts. EPHA2 immunolabeling showed intracellular accumulation of the mutant EPHA2-β-galactosidase fusion protein that induced a cytoprotective ER stress response and in *Epha2^+/^^−^* lenses was also accompanied by glutathione redox imbalance.

**Conclusions:**

Both, *Epha2^−^^/^^−^* and *Epha2^+/^^−^* mice develop age-related cortical cataract; age as a function of *Epha2* genotype, sex, and genetic background influence *Epha2*-related cataractogenesis in mice.

Age-related cataract generally occurs after the age of 45 years due to progressive accumulation of insults in the ocular lens,^1^ and accounts for approximately 48% of blindness worldwide.^2^ It is a multifactorial disease with an interplay of environmental and genetic factors contributing to the disease risk. Older age, female gender, lifestyle choices, such as smoking and alcohol consumption, exposure to ultraviolet radiation from sunlight, corticosteroid use, and diseases, such as diabetes, pseudoexfoliation syndrome, and myopia, increase the risk of cataract.^2^^–^^8^ Depending upon the location of lens opacity, the disease presents as nuclear, cortical, and posterior subcapsular cataract, or mixed cataract involving more than one of these subtypes. Several epidemiological studies have shown ethnic differences in the prevalence of age-related cataract subtypes, likely due to differences in lifestyle, climatic environment, and genetic background.^9^^–^^14^ Genetic predisposition to cataract development has been evidenced through twin studies and clustering of the disease within families.^15^^–^^17^ Single nucleotide polymorphisms (SNPs) in multiple genes have been associated with increased susceptibility to age-related cataract.^18^^–^^28^

To date, genetic variation in the *EPHA2* gene is the only genetic factor that has been reproducibly associated with the risk of age-related cataract in multiple geographically and ethnically diverse populations.^29^^–^^32^ SNPs in and around the *EPHA2* gene are associated with the risk of all subtypes of the disease. In addition, a mis-sense coding variant in this gene causes Mendelian age-related cortical cataract with incomplete penetrance.^33^ Protein truncating and mis-sense mutations in the *EPHA2* gene have been implicated in congenital cataract.^33^^–^^36^

*EPHA2* encodes a membrane protein of the Eph tyrosine kinase receptor family that binds to membrane bound ephrin ligands on neighboring cells and leads to bidirectional signaling.^37^ EPHA2 signaling plays a role in epithelial homeostasis during development and adulthood.^30^^,^^38^^,^^39^ Interestingly, *Epha2*-null mice progressively develop cortical cataract with aging.^30^ The loss of EPHRIN-A5, a disputed ligand of EPHA2 in the lens, also leads to age-related cataract in mice.^40^^,^^41^ Thus, EPHA2 signaling is pivotal for mammalian lens development and for maintaining lens transparency throughout life.

Age-related cataract has been reported only in *Epha2*-null mice; mice carrying a single functional allele of *Epha2*, heterozygous-null, were not found to develop cataract up to 14 months of age.^30^ Whether *Epha2* heterozygous-null mice develop cataract at a later age is not known. Interestingly, biological factors, such as age and female gender, that increase the risk of age-related cataract,^6^^,^^8^^,^^42^^,^^43^ also influence *EPHA2* expression and/or signaling; EPHA2 expression reduces in mouse lens with age,^30^ and in cultured mammary epithelial cells, estrogen negatively regulates EPHA2 signaling.^44^ Hence, we hypothesized that biological factors that increase the risk of age-related cataract, particularly, age, genetic background, and sex, interact with EPHA2 signaling and influence cataract development, leading to cataract with increase in age in *Epha2* heterozygous-null mice. In the present study, we tested this hypothesis in *Epha2-*knockout mice and found that age as a function of *Epha2* genotype, genetic background, and sex significantly influence *Epha2*-related cataract development.

## Materials and Methods

### Animal Maintenance and Breeding

All experimental procedures on animals conformed to the ARVO Statement for the Use of Animals in Ophthalmic and Vision Research and were approved by the Animal Welfare Committee, Flinders University, South Australia.

The *Epha2*-knockout mice (B6; 129P2-*Epha2^Gt(KST085)Byg^*; KST085) on C57BL/6J background, generated using the secretory gene trapping method,^45^^,^^46^ were purchased from Mutant Mouse Regional Resources Centers, Missouri, USA. The anterior subcapsular cataract-causing deletion mutation in the *Beaded filament structural protein 2* (*Bfsp2)* gene present in the 129P2 strain^47^^,^^48^ was eliminated by selecting no mutation carriers as breeders for colony maintenance, as identified by polymerase chain reaction (PCR)-based *Bfsp2* genotyping in the first two generations. As the *Epha2*-knockout mice carry *Epha2* gene-trapped allele/s, the symbols *Epha2^+/^*^−^ and *Epha2^−^^/^^−^* used hereafter represent heterozygous and homozygous *Epha2* gene-trap allele/s, respectively. To generate *Epha2*-knockout mice on FVB:C57BL/6J (50:50) mixed background, a homozygous *Epha2*-knockout (*Epha2^−^^/^^−^*) mouse on C57BL/6J background was crossed with a wild-type FVB/NJ mouse, and three pairs of resulting heterozygous *Epha2*-knockout (*Epha2^+/^^−^*) F2 mice on mixed background were interbred for colony maintenance. *Epha2^+/+^*, *Epha2^+/^*^−^, and *Epha2^−^^/^^−^* littermates from subsequent generations of these mice were used for experiments.

### Genotyping

The FVB/NJ strain used for generation of *Epha2*-knockout mice on mixed background also carries the anterior subcapsular cataract causing mutation in the *Bfsp2* gene.^47^^,^^48^ Hence, all mice on mixed background used in this study were genotyped for that and the *Epha2* knockout mutation. *Bfsp2* and *Epha2* genotyping was performed as previously described,^49^ except for genotyping *Bfsp2* mutation in some mice on mixed background mutant reverse primer 5′AGGGAGATCCTCTTGCTATCTAGCT was used, and PCR performed with annealing at 62°C for 40 cycles.

### Ophthalmic Examination

Ophthalmic examination of the lens was performed in anesthetized mice on a photo-slit lamp biomicroscope (Topcon Medical Systems Inc., Oakland, NJ, USA) as previously described;^49^ cortical cataract was graded using Lens Opacities Classification System (LOCS) III in each eye of an animal.^50^ In mice on mixed background, anterior subcapsular cataract was also graded using the grading system similar to that used previously.^49^ The observations were recorded by digital photography at 40 × magnification and animals revived after examination as previously described.^49^

### Histological Analysis

Mice were euthanized and whole eyes enucleated, fixed in 10% neutral buffer formalin, and paraffin embedded for histology. Histological analysis and imaging, using a 40 × objective, were performed as previously described.^49^^,^^51^

### Immunolabeling

Antigens were retrieved by heat-induced epitope retrieval in 0.01 M citrate buffer with 0.05% Tween 20, pH 6 for N-cadherin and β-catenin and in Target Retrieval Solution, pH 9 (Dako Australia Pty Ltd., New South Wales, Australia) for EPHA2 labeling, as described elsewhere.^51^ Sections were blocked with 3% goat or donkey serum (Sigma-Aldrich Pty. Ltd., Sydney, Australia), incubated with mouse anti-N-cadherin (1:200; Life Technologies Australia Pty. Ltd., Victoria, Australia) or mouse anti-β-catenin (1:200; BD Transduction Laboratories, San Diego, CA, USA) or goat anti-mouse (m) EPHA2 (1:40; R&D Systems, Inc., Minneapolis, MN, USA) primary antibody followed by goat anti-mouse or donkey anti-goat IgG Alexa Fluor 488-conjugated (1:1000; Life Technologies Australia Pty. Ltd.) secondary antibody; control sections were incubated with equivalent amount of mouse or goat IgG. Labeled sections were mounted in Prolong AntiFade with DAPI (Life Technologies Australia Pty. Ltd.) and imaged as previously described.^52^

### Western Blotting

Mice were euthanized and ocular lenses dissected and snap frozen for later protein extraction. For protein extraction, whole lens tissue was homogenized in 50 or 100 µl RIPA buffer,^53^ incubated on ice for 30 minutes, centrifuged to remove insoluble fraction, and clear lysate used for analysis. Forty microgram of lens protein per sample was size-separated by SDS-PAGE on 4 to 20% gradient Mini-PROTEAN TGX Stain-Free pre-cast gel (BioRad Laboratories Pty. Ltd., New South Wales, Australia), and proteins transferred onto polyvinylidene fluoride-low fluorescence (PVDF-LF) membrane in Trans-Blot Turbo buffer (BioRad Laboratories Pty. Ltd.) with 15% or 20% methanol on a Trans-Blot Turbo Transfer System (BioRad Laboratories Pty. Ltd.) as per the manufacturer's protocols. Protein gel and blot were imaged on a Chemi Doc Touch Imaging System (BioRad Laboratories Pty. Ltd.) to monitor protein transfer and document protein loading. Western blots were incubated with rabbit anti-β-galactosidase (1:500; Novus Biologicals, LLC, Centennial, CO, USA) or rabbit anti-BiP (1:1000; Cell Signaling Technology, Danvers, MA, USA) or rabbit anti-ATF6 (1:1000; Sigma-Aldrich) or mouse anti-CHOP (1:1000; Cell Signaling Technology) primary antibody followed by donkey anti-rabbit IgG-horseradish peroxidase conjugated (1:1000; Cat #711-035-152, Jackson ImmunoResearch Laboratories, Inc., West Grove, PA, USA) or donkey anti-mouse IgG-horseradish peroxidase conjugated (1:1000; Cat #715-35-150, Jackson ImmunoResearch Laboratories) secondary antibody. Antibody binding was detected with Clarity Western ECL Substrate (BioRad Laboratories Pty. Ltd.) and imaged as described above and following the manufacturer's protocol.

### Xbp1 mRNA Splicing

Total RNA from both lenses of a mouse was extracted, and cDNA reverse transcribed (RT) from 0.74 µg RNA, as previously described.^49^ *Xbp1* transcript was amplified from cDNA by PCR using gene-specific primers^54^ and Hot Star Taq *Plus* DNA polymerase (Qiagen Pty. Ltd., Victoria, Australia) with Q solution. PCR conditions included enzyme activation at 95°C for 5 minutes, followed by 35 or 37 cycles of denaturation at 95°C, annealing at 62°C, and elongation at 72°C for 30 seconds each, and a final elongation at 72°C for 5 minutes. Amplicons were cleaned with Wizard SV Gel and PCR Clean-up System (Promega Australia, New South Wales, Australia), *PstI* digested and analyzed by agarose gel electrophoresis. Band intensities were quantified using the ImageJ software. Percent amount of spliced *Xbp1* mRNA was assessed as described elsewhere.^54^ Specificity of amplicons was confirmed by sequencing.

### Bip mRNA Expression

Total RNA was extracted, and cDNA synthesized as described above. From cDNA, *Bip* transcript was amplified by PCR using gene-specific primers 5′TGTGTGAGACCAGAACCGTC (forward) and 5′TCGCTGGGCATCATTGAAGT (reverse), and Hot Star Taq *Plus* DNA polymerase with Q solution. PCR was performed with enzyme activation at 95°C for 5 minutes, followed by 30 cycles of denaturation at 95°C for 30 seconds, annealing at 62°C for 30 seconds and elongation at 72°C for 45 seconds, and a final elongation at 72°C for 5 minutes. *B2m* transcript was amplified, to use as normalization control, as previously described,^49^ except amplification was performed for 39 cycles. Amplicons were analyzed by agarose gel electrophoresis and band intensities quantified using the ImageJ software. Specificity of amplicons was confirmed by sequencing.

### Chromatography

Mouse lenses were immediately snap-frozen after dissection for later analysis. The lenses were homogenized as described in the Supplementary Methods. Free glutathione (GSH), glutathione disulfide (GSSG), and total soluble GSH were determined using the method described by Altes et al.^55^ with slight modification (i.e. inclusion of *N*-acetylcysteine as an internal standard). Concentration of protein-bound GSH (PSSG) was estimated as described in the Supplementary Methods. Protein concentration was determined using the Bradford method.^56^ The calibration curve used for determination of free GSH, GSSG, and total GSH is included in Supplementary Figure S1, and for determination of PSSG in Supplementary Figure S2. Levels of GSH and GSSG are reported as nmol/mg of protein, and of PSSG as nmol/mg of dried tissue.

### Statistical Analysis

Statistical analyses of cataract grading data were performed using IBM Statistical Package for the Social Science (SPSS) 19. Anterior cortical cataract (ACC) grade in each animal was calculated as an average of cataract grades in the two eyes. Data for each group are presented as mean cortical cataract grade. Effect of *Epha2* genotype at an age and of sex on cataract progression were analyzed using the Kruskal-Wallis test and significant differences analyzed pairwise using the Mann-Whitney *U* test. Effect of age on cataract progression in a genotype was analyzed using the Friedman test, and any significant differences further analyzed by the Wilcoxon Signed Ranks test. Statistical analyses of *Xbp1* mRNA splicing and chromatography data were performed using GraphPad Prism 8 (version 8.4.0; GraphPad Software, San Diego, CA, USA). Differences in *Xbp1* mRNA splicing between genotypes were compared by ANOVA with Dunnett's T test for multiple comparisons. Each parameter estimated by chromatography was compared between two genotypes by two-tailed *t*-test. Significance level was set at 0.05 or as indicated.

## Results

### Age and Genotype Influence *Epha2*-related Cataract Development

**Figure 1.**
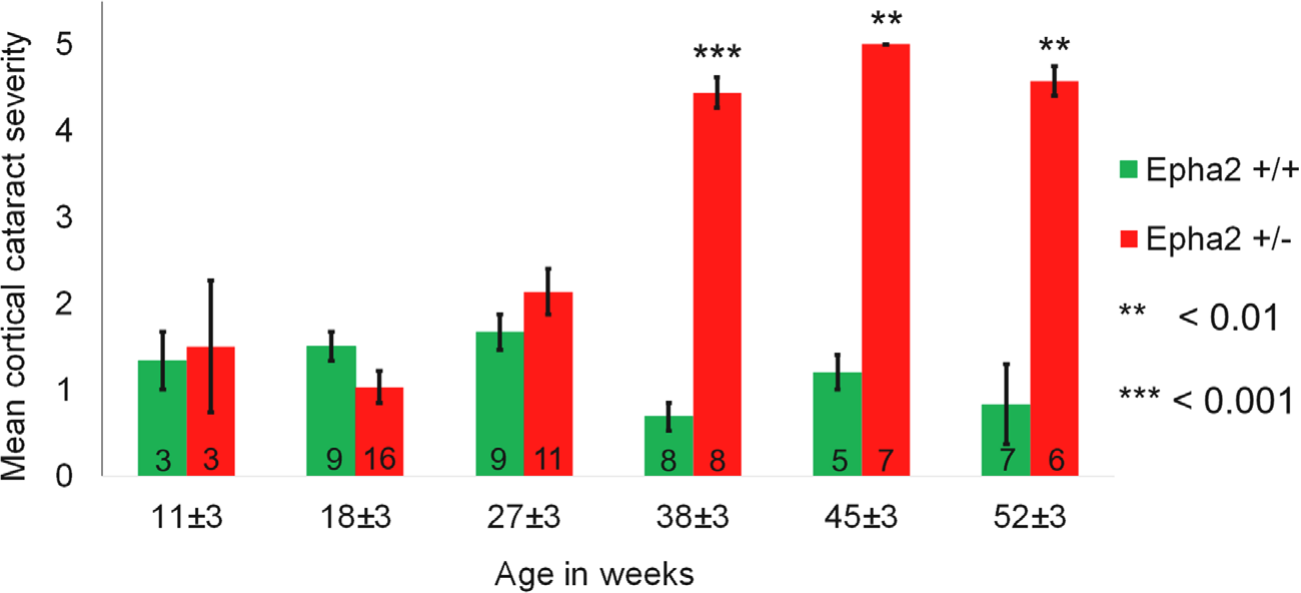
**Comparison of anterior cortical lens opacity in *Epha2^+/+^* and *Epha2^+/^^−^* mice of different ages.** Anterior cortical cataract was examined in groups of 11, 18, 27, 38, 45, and 52-week-old *Epha2^+/+^* and *Epha2^+/^^−^* mice on C57BL/6J background. The bars represent mean cortical cataract grade in each age group. Data from each age group was compared by Mann-Whitney *U* test. Significant *P* values are indicated. The group sizes are indicated in the bars. Error bars represent standard error of the mean.

In order to determine whether *Epha2* heterozygous-null mice develop cataract with increase in age, different groups of 11, 18, 27, 38, 45, and 52 weeks old *Epha2* wild-type (*Epha2^+/+^*) and *Epha2^+/^^−^* mice on C57BL/6J background were examined for lens opacity. *Epha2^+/+^* and *Epha2^+/^^−^* 11, 18, and 27-week-old mice exhibited similar mild anterior cortical lens opacity (Fig. 1, Supplementary Table S1) whereas 38, 45, and 52-week-old *Epha2^+/^^−^* mice exhibited significantly severe anterior cortical opacities (grade 4 or 5) than age-matched *Epha2^+/+^* mice (38 weeks, *P* < 0.001; 45 and 52 weeks, *P* < 0.01; see Fig. 1, Supplementary Table S1), indicating development of ACC. As mild lens opacity seen in *Epha2^+/+^* mice of all ages did not progress with age and was reversible, we concluded that it was transiently induced by anesthesia, as reported elsewhere.^57^

**Figure 2.**
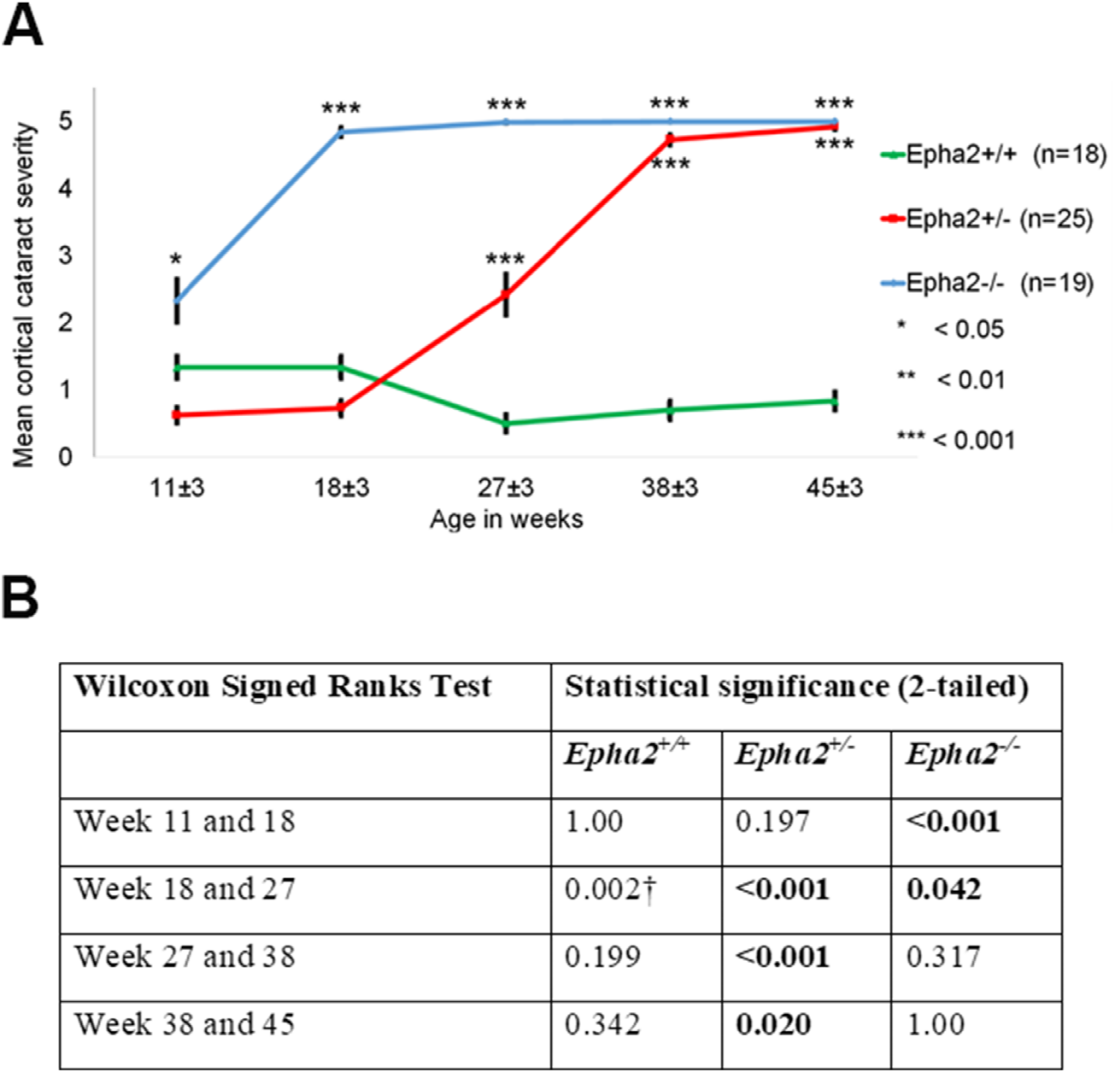
**Progression of anterior cortical cataract in *Epha2-*knockout mice.***Epha2^+/+^*, *Epha2^+/^^−^*, and *Epha2^−^^/^^−^* mice on C57BL/6J background were monitored for cataract development from 11 ± 3 weeks to 45 ± 3 weeks of age. (**A**) Mean cortical cataract grade in each genotype of mice at each time point has been graphically represented. *Epha2^−^^/^^−^* mice developed severe cortical cataract at 18 ± 3 weeks of age and *Epha2^+/^^−^* mice at 38 ± 3 weeks of age. *Epha2^+/+^* mice presented with very mild lens opacity up to 45 ± 3 weeks of age. The Mann Whitney *U* test *P* values from comparison with *Epha2^+/+^* mice are indicated. Error bars represent standard error of the mean. After an animal exhibited a cataract grade of 4 or 5, the same grade was attributed to subsequent time points for data analysis, assuming no further increase in cataract severity occurred. Missing data at the first time point was substituted with data from the second time point assuming lesser cataract severity at the first than second time point. (**B**) The table shows Wilcoxon Signed Ranks test *P* values of comparison between time points indicating the effect of age on cataract progression in each genotype of mice. † Indicates likely artifact of anesthetic-induced cataract.

**Figure 3.**
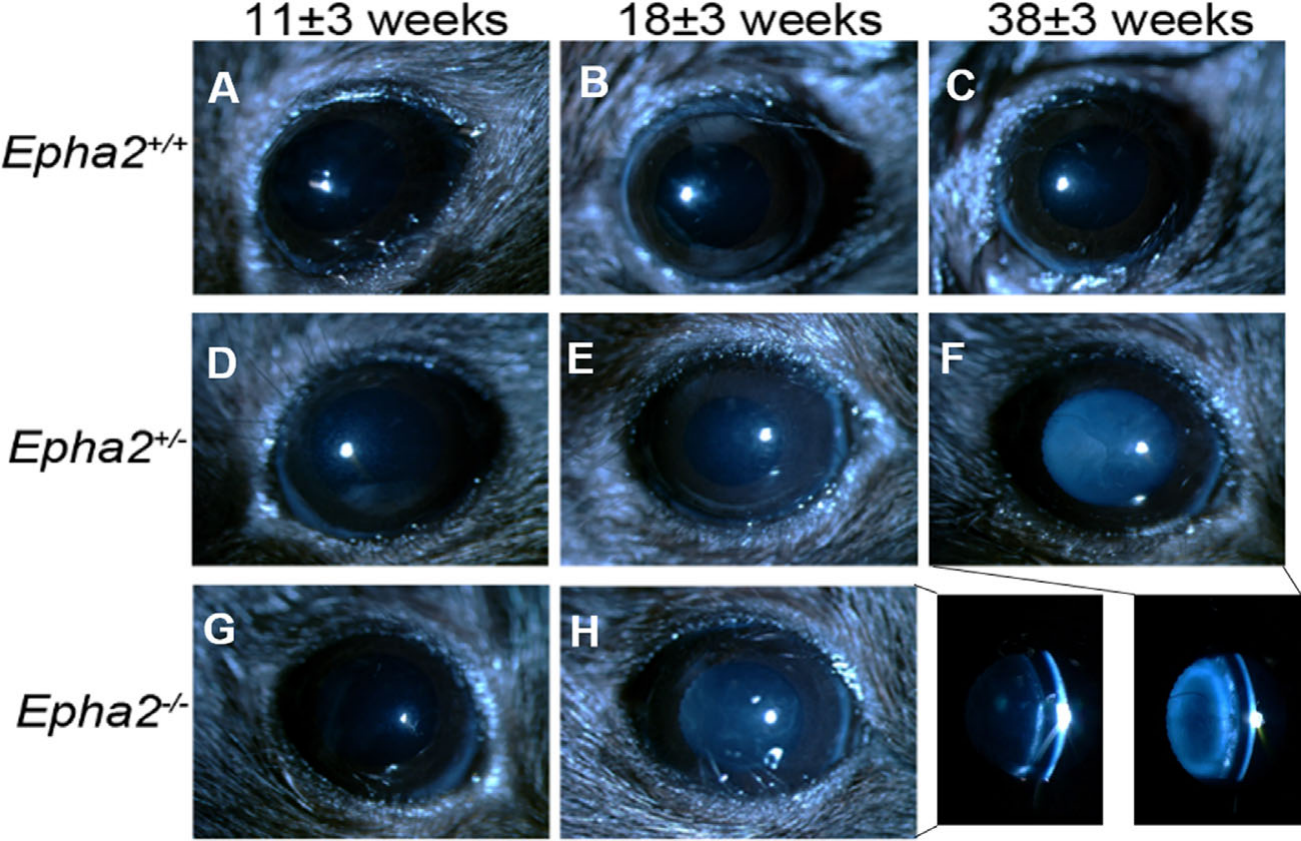
**Anterior cortical cataract developed in *Epha2-*knockout mice.** Representative ophthalmic images of *Epha2^+/+^* (**A, B,** **C**) and *Epha2^+/^^−^* (**D, E,** **F**) mice at 11 ± 3, 18 ± 3, and 38 ± 3 weeks of age and *Epha2^−^^/^^−^* (**G,** **H**) mice at 11 ± 3 and 18 ± 3 weeks of age, on C57BL/6J background, are shown. *Epha2^−^^/^^−^* mice progressively developed severe ACC by 18 ± 3 weeks of age, panel **H**, and *Epha2^+/^^−^* mice by 38 ± 3 weeks of age, panel **F**. The bottom right panel shows cross-section illumination images of cortical cataracts seen in panels **H** and **F**. Lenses of *Epha2^+/+^* mice remained relatively clear up to 38 ± 3 weeks of age, panel **C**, and later (not shown). Magnification, times 40.

The phenotype of cataract observed in *Epha2^+/^^−^* mice was consistent with that reported in *Epha2^−^^/^^−^* mice in previous studies,^30^^,^^39^ and manifestation of severe cataract in 38-week-old and older mice supported our hypothesis. This finding is significantly different than no cataract reported in a previous study in *Epha2^+/^^−^* mice carrying the same knockout mutation on FVB background.^30^ Thus, to confirm this finding, in an independent experiment, we determined progression of cataract in cohorts of *Epha2^+/+^*, *Epha2^+/^^−^*, and *Epha2^−^^/^^−^* mice (*n* ≥ 18 per group) on C57BL/6J background from 11 to 45 weeks of age or until severe cataract developed (grade 4 to 5). Examinations were performed at approximately eight weekly intervals. *Epha2^+/+^* mice exhibited anesthetic-induced mild ACC (grade < 2) up to 45 weeks of age. In comparison, *Epha2^+/^^−^* mice presented with mild ACC up to 18 weeks, moderate ACC by 27 weeks (grade >2, *P* < 0.001) and severe ACC by 38 or 45 weeks of age (grade 4 or 5, *P* < 0.001; Figs. 2A, 3, Supplementary Table S2). The differences in severity of cataract in *Epha2^+/^^−^* mice between different ages were statistically significant (Fig. 2B). In *Epha2^−^^/^^−^* mice, moderately severe ACC was observed earlier, at 11 weeks of age (grade >2, *P* < 0.05), and severe ACC by 18 or 27 weeks of age (grade 4 or 5, *P* < 0.001), compared to *Epha2^+/+^* mice (see Figs. 2A, 3, Supplementary Table 2). The severity of cataract in these mice differed significantly between ages (see Fig. 2B). The observed differences in cataract severity between *Epha2^+/+^*, *Epha2^+/^^−^*, and *Epha2^−^^/^^−^* mice persisted up to 45 weeks of age (*P* < 0.001; see Fig. 2A). Overall, these experiments showed that *Epha2^+/^^−^* mice also develop age-related cataract and revealed a significant interactive effect of age and *Epha2* genotype on cataract development in *Epha2-*knockout mice on C57BL/6J background.

### Lens Architecture Changes With Age and Genotype in *Epha2*-Knockout Mice

**Figure 4.**
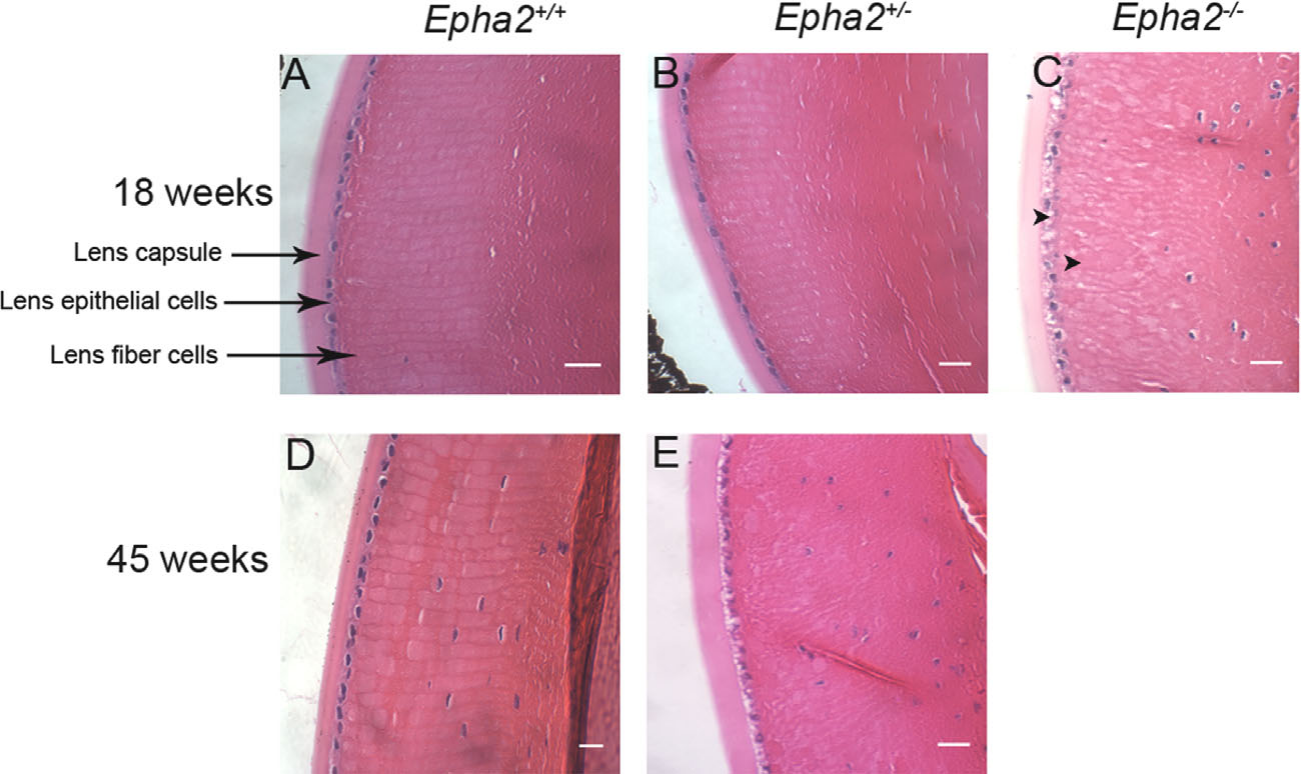
**Histological analysis showing disruption of lens architecture in *Epha2-*knockout mouse lenses.** Representative images of hematoxylin and eosin-stained sections of lenses of 18-week-old *Epha2^+/+^* (**A**), *Epha2^+/^^−^* (**B**), and *Epha2^−^^/^^−^* (**C**) mice and 45-week-old *Epha2^+/+^* (**D**) and *Epha2^+/^^−^* (**E**) mice on C57BL/6J background showing nuclei (*blue*) and cytoplasm (*pink*) of lens epithelial and fiber cells as well as lens capsule. Disruption of fiber cell arrangement in the lens cortex can be seen in 18-week-old *Epha2*^−/−^, **C**, and 45-week-old *Epha2*^+/−^, **E**, mouse lenses as opposed to arrangement in meridional rows in *Epha2*^+/+^, **A** and **D**, and 18-week-old *Epha2*^+/−^ lenses, **B**. *Arrowheads* show presence of vacuoles in the lens cortex and lens epithelial cells. Nucleated fiber cells are visible in those sections closer to the lens equator. Scale-bars 20 µm.

Histological analysis of 18-week-old *Epha2^+/+^* and *Epha2^+/^^−^* lenses showed normal lens epithelial and fiber cell arrangement (Figs. 4A, 4B) whereas age-matched *Epha2^−^^/^^−^* lenses showed grossly disorganized, irregularly shaped, and swollen fiber cells and presence of vacuoles in the lens epithelium (arrowheads, Fig. 4C). Similar disruption of lens architecture was observed in 45-week-old *Epha2^+/^^−^* compared to age-matched *Epha2^+/+^* lenses (Figs. 4D, 4E). The histological observations were consistent with ophthalmic observations in all three genotypes.

**Figure 5.**
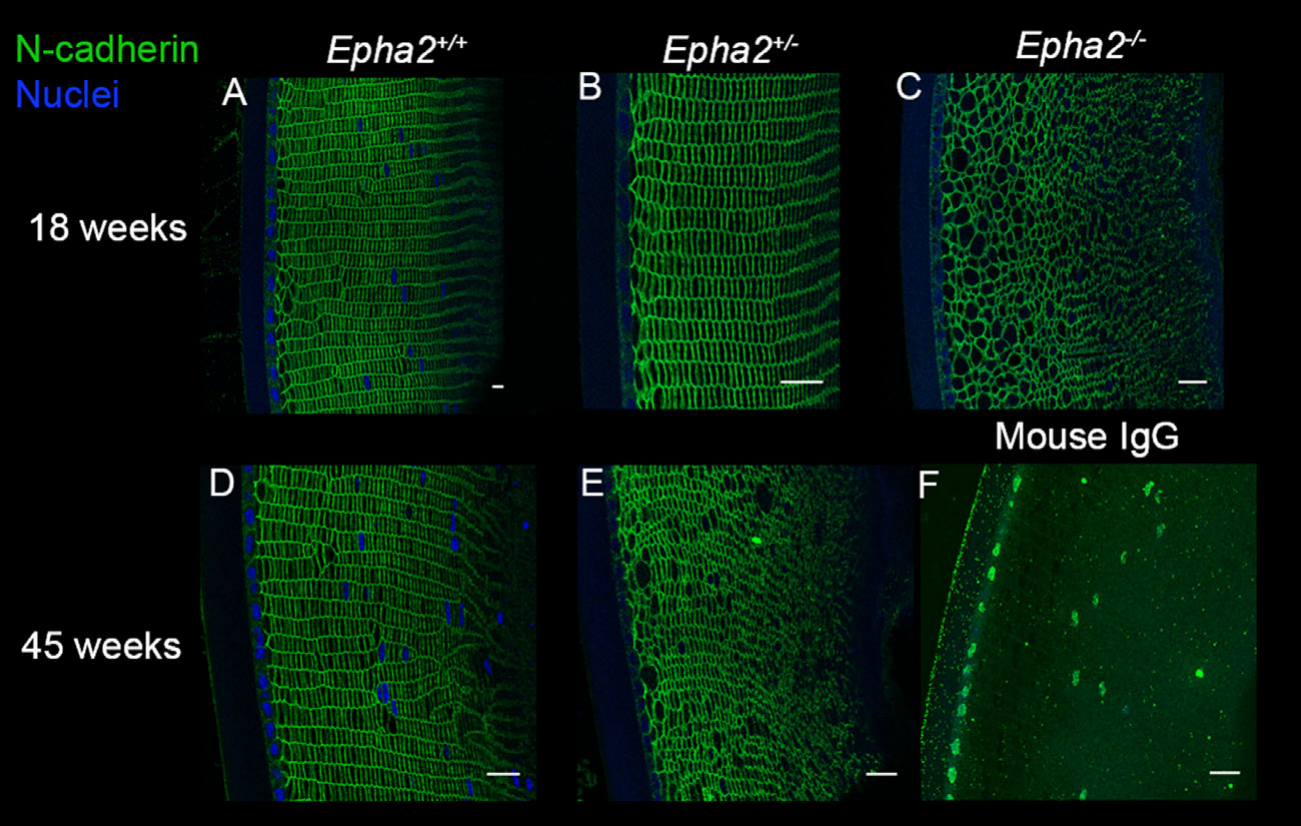
**N-cadherin immunolabeling showing disorganization of cellular architecture in *Epha2-*knockout mouse lenses.** Representative confocal microscopy images of sections of lenses of 18-week-old *Epha2^+/+^* (**A**), *Epha2^+/^^−^* (**B**), and *Epha2^−^^/^^−^* (**C**), and 45-week-old *Epha2^+/+^* (**D**) and *Epha2^+/^^−^* (**E**) mice on C57BL/6J background immunolabeled with mouse anti-N-cadherin antibody (*green*) and DAPI (*blue*) to label the nuclei are shown. N-cadherin labeling delineated the cell boundary and showed well packed fiber cells arranged in meridional rows in 18, **A**, and 45-week-old *Epha2*^+/+^, **D**, and 18-week-old *Epha2*^+/−^, **B**, lenses. In contrast, it showed larger and disorganized fiber cells in 18-week-old *Epha2*^−/−^, **C**, and 45-week-old *Epha2*^+/−^, **E**, lenses. Sections probed with mouse IgG (negative control) revealed little or no non-specific labeling (**F**). Scale-bars 20 µm.

**Figure 6.**
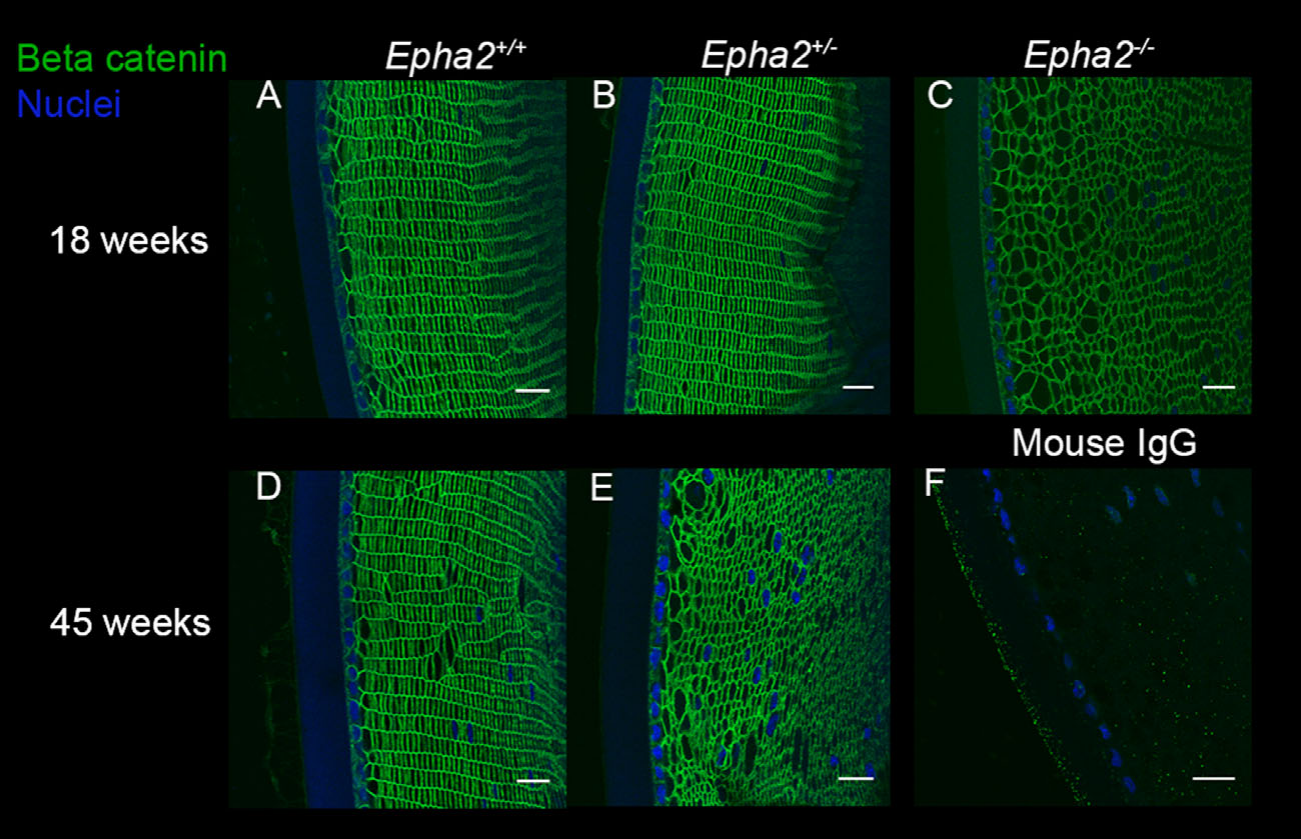
**β-Catenin labeling showing breakdown of lens architecture in *Epha2-*knockout mouse lenses.** Representative confocal microscopy images of sections of lenses of 18-week-old *Epha2^+/+^* (**A**), *Epha2^+/^^−^* (**B**), and *Epha2^−^^/^^−^* (**C**) and 45-week-old *Epha2^+/+^* (**D**) and *Epha2^+/^^−^* (**E**) mice on C57BL/6J background immunolabeled with mouse anti-β-catenin antibody (*green*) and DAPI (*blue*) to stain the nuclei are shown. Similar to N-cadherin labeling, β-catenin labeling delineated meridional rows of fiber cells in 18, A, and 45-week-old *Epha2*^+/+^, **D**, and 18-week-old *Epha2*^+/−^, **B**, lenses. In contrast, gross disorganization of fiber cells in 18-week-old *Epha2*^−/−^, **C**, and 45-week-old *Epha2*^+/−^, **E**, lenses was observed. Sections probed with mouse IgG (negative control) revealed little or no non-specific labeling (**F**). Scale-bars 20 µm.

To determine the cellular organization and cell-cell junction integrity, lens sections of *Epha2^+/+^*, *Epha2^+/^^−^*, and *Epha2^−^^/^^−^* mice were labeled for adherens junction protein N-cadherin and adherens junction associated protein β-catenin^58^^,^^59^ at 18 and 45 weeks of age, when *Epha2^−^^/^^−^* and *Epha2^+/^^−^* mice, respectively, develop severe cataract. N-cadherin primarily showed peripheral localization in cortical lens fiber cells in lenses of all three genotypes of mice at both the ages (Fig. 5). In addition, it revealed regular arrangement of fiber cells in meridional rows in lenses of *Epha2^+/+^* mice at both the ages and 18-week-old *Epha2^+/^^−^* mice (see Figs. 5A, 5B, 5D). However, disorganized, irregularly shaped, and sized fiber cells were observed in 18-week-old *Epha2^−^^/^^−^* and 45-week-old *Epha2^+/^^−^* mouse lenses (see Figs. 5C, 5E). Similar results were obtained upon immunolabeling of β-catenin (Fig. 6), demonstrating correlation between loss of lens fiber cell organization and development of severe cataract in both, *Epha2^−^^/^^−^* and *Epha2^+/^^−^* mice.

**Figure 7.**
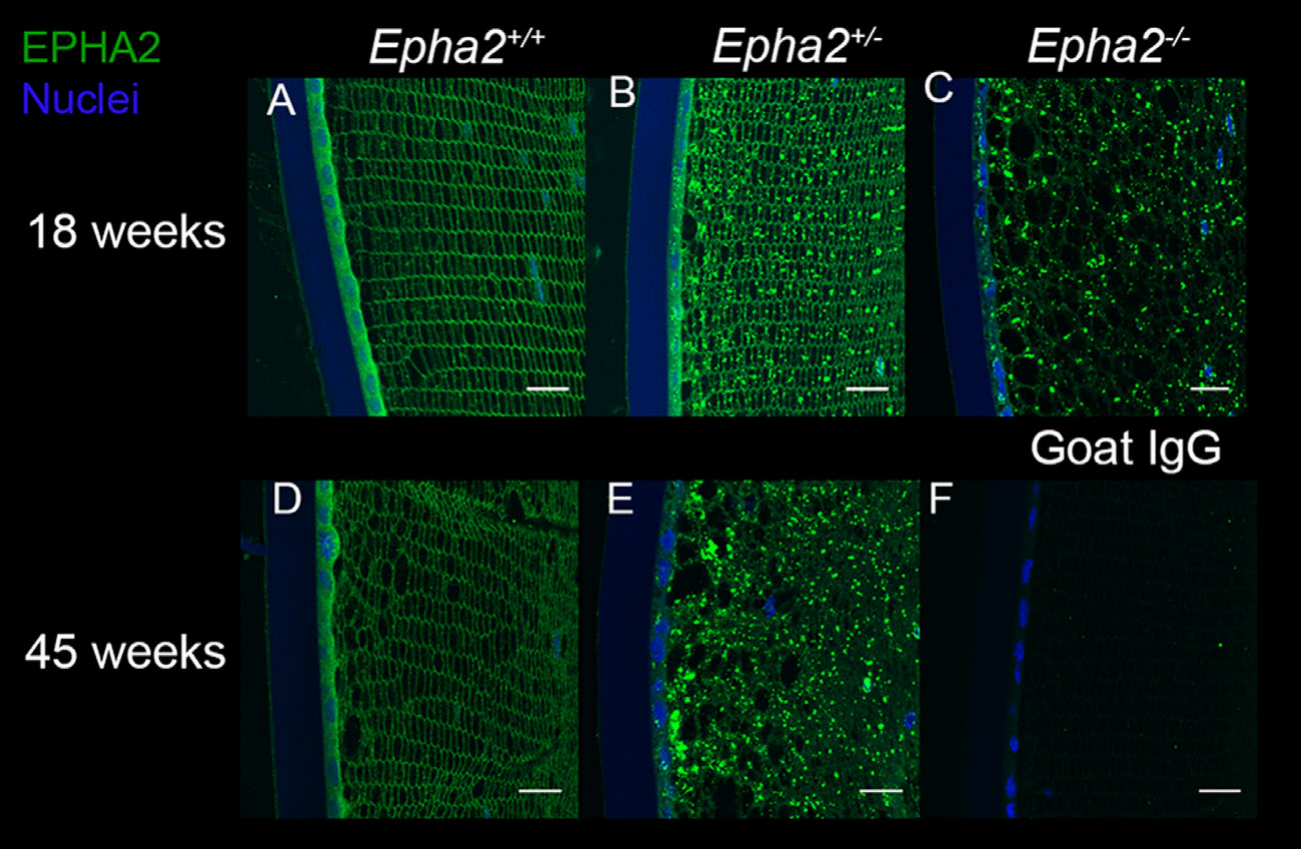
**Accumulation of mutant EPHA2 protein in *Epha2-*knockout mouse lenses detected by immunolabeling.** Representative confocal microscopy images of lens sections of 18-week-old *Epha2^+/+^* (**A**), *Epha2^+/^^−^* (**B**), and *Epha2^−^^/^^−^* (**C**) and 45-week-old *Epha2^+/+^* (**D**) and *Epha2^+/^^−^* (**E**) mice on C57BL/6J background were immunolabeled with anti-mouse EPHA2 antibody (*green*) and DAPI (*blue*) to stain the nuclei. The lenses of 18, **A**, and 45-week-old *Epha2*^+/+^, **D**, showed the EPHA2 protein in lens epithelial cells and at the lens fiber cell periphery. Eighteen-week-old lenses of *Epha2*^+/−^ mice, **B**, in addition to the protein in lens epithelium and lens fiber cell periphery, exhibited its presence in a granular pattern in cells. Lenses of 45-week-old *Epha2*^+/−^, **E**, and 18-week-old *Epha2*^−/−^, **C**, mice showed presence of the protein only in granular pattern likely indicating accumulation of the partial EPHA2-β-galactosidase fusion protein in lens cells. Similar labeling was absent in negative control sections incubated with goat IgG (**F**). Scale-bars 20 µm.

As the *Epha2*-knockout mouse strain used for this study was generated by secretory gene trapping approach and expresses partial EPHA2 ectodomain fused to β-galactosidase reporter protein that is potentially trapped in inclusion bodies inside the cell,^45^^,^^46^ we determined subcellular localization of EPHA2 (wild-type or fusion protein) at 18 and 45 weeks of age. Immunolabeling showed the presence of wild-type EPHA2 in the lens epithelium and at cell membrane in fiber cells in *Epha2^+/+^* mice at both the ages (Figs. 7A, 7D), and in agreement with arrangement of lens fiber cells in meridional rows seen by N-cadherin and β-catenin labeling. The localization pattern was consistent with that reported previously^30^^,^^38^ and correlated with the role of EPHA2 in maintenance of inter-cellular contacts. Interestingly, in lenses of 18-week-old *Epha2^+/^^−^* mice, along with labeling of the cell membrane, EPHA2-positive labeling was observed in a granular pattern in lens epithelial and fiber cells (Fig. 7B). In lenses of 45-week-old *Epha2^+/^^−^* and 18-week-old *Epha2^−^^/^^−^* mice, mainly granular labeling pattern was observed (Figs. 7C, 7E). The granular labeling pattern revealed by an antibody that recognizes the EPHA2 ectodomain is consistent with intracellular trapping of the partial EPHA2-β-galactosidase fusion protein in the lens cortex. Similar granular labeling pattern was observed in lenses of 18-week-old *Epha2^−^^/^^−^* and 18 and 45-week-old *Epha2^+/^^−^* mice upon labeling of β-galactosidase reporter protein (Supplementary Fig. S3). However, fewer granules were present in 18-week-old compared to 45-week-old *Epha2^+/^^−^* lenses, as seen with EPHA2 labeling.

**Figure 8.**
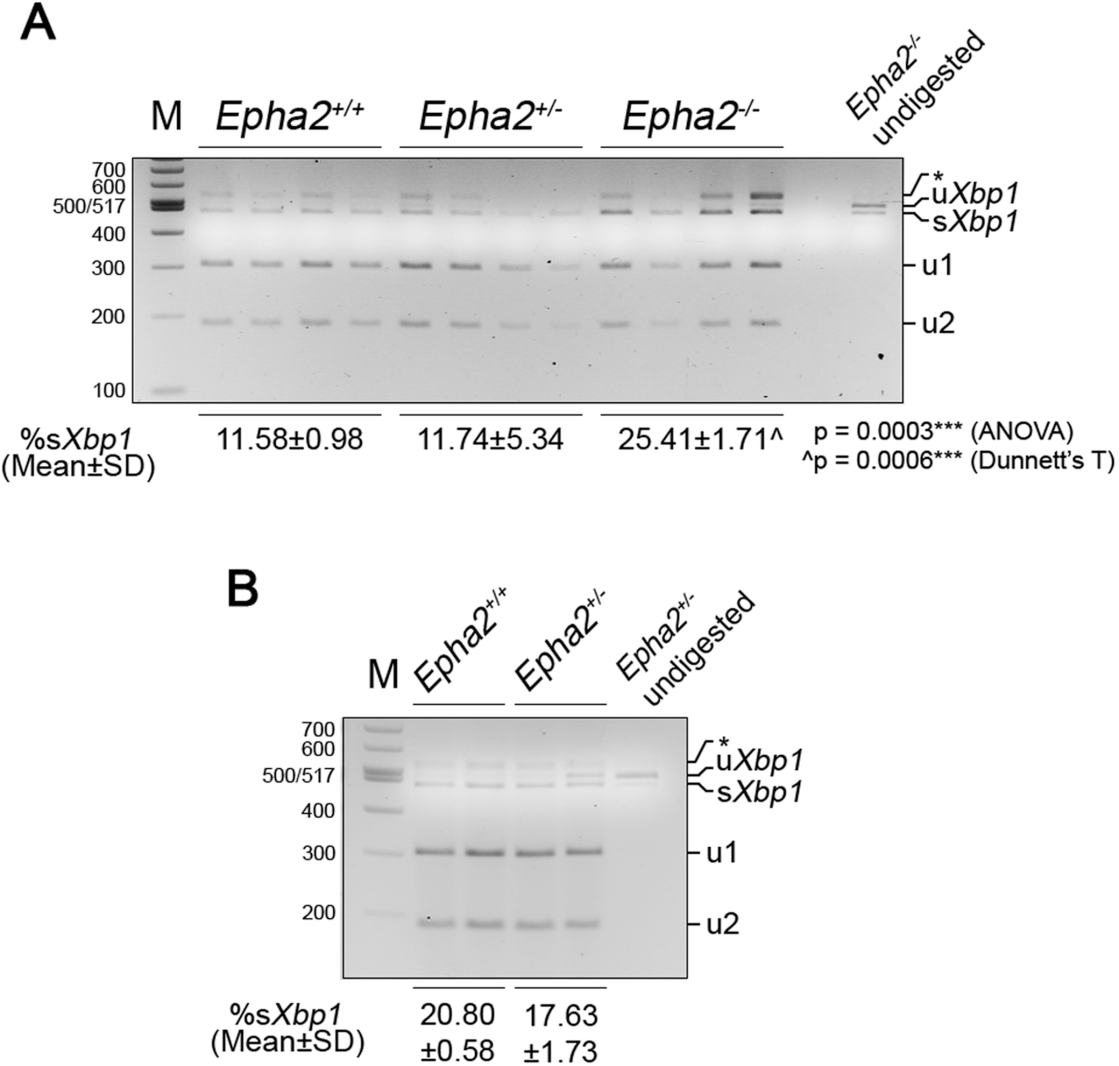
**Assessment of *Xbp1* mRNA splicing in *Epha2*-knockout mouse lenses.** Partial *Xbp1* mRNA flanking the 26-nucleotide long intron was amplified from lenses (*n* = 4) of 8-week-old *Epha2^+/+^*, *Epha2^+/^^−^*, and *Epha2^−^^/^^−^* (**A**), and 14 to 19-week-old *Epha2^+/+^* and *Epha2^+/^^−^* (**B**), mice on C57BL/6J background by RT-PCR using gene-specific primers. The PCR product was digested with *Pst*I restriction enzyme and size separated on a 3% agarose gel. In **B**, each lane represents analysis of lenses of two mice of the same sex. *Pst*I digestion of the unspliced variant (u*Xbp1*) at the *Pst*I site in the intron resulted in 301 bp (u1) and 193 bp (u2) fragments. The spliced variant (s*Xbp1*) generated by excision of the intron, as expected, was not digested by *Pst*I. Both unspliced and spliced variants were detected in lenses of all genotypes and at both the ages, **A** and **B**. *Asterisks* marks a hybrid of the two variants. The proportion of the spliced variant in a sample was calculated (sXbp1/(uXbp1+sXbp1+u1+u2)). Mean percent spliced variant in lenses of each genotype is indicated; SD = standard deviation. The data in **A** was statistically analyzed by ANOVA with Dunnett's T test for multiple comparisons. ***p < 0.001; ^ versus *Epha2^+/+^*.

Next, to investigate if accumulation of the EPHA2-β-galactosidase fusion protein in lens cells led to endoplasmic reticulum (ER) stress and unfolded protein response (UPR), we examined expression of the ER stress chaperone, BiP.^60^ Upon analysis of lens proteins from 18, 27, and 44 week-old *Epha2^+/+^*, *Epha2^+/^^−^*, and *Epha2^−^^/^^−^* mice by Western blotting, BiP expression was not detected in any genotype at any age but was readily detected in HeLa cell lysate used as positive control (Supplementary Fig. S4A). Undetectable BiP expression in wild-type lenses is consistent with expression being restricted to the lens epithelium and newly differentiating fiber cells, as reported previously.^61^^,^^62^ In order to determine whether ER stress and UPR is induced earlier than 18 weeks, we examined expression of BiP, as well as of transcription factors XBP1 and ATF6, activated by ER stress, and CHOP, a downstream effector of UPR,^60^ in lenses of 8-week-old *Epha2^+/^^−^* and *Epha2^−^^/^^−^* and age-matched *Epha2^+/+^* mice. Expression of BiP, ATF6, and CHOP was analyzed by Western blotting and of *Xbp1* by RT-PCR; *Bip* expression was also analyzed by RT-PCR. Once again, expression of BiP protein was not detected in lenses of any genotype but was detected in the HeLa cell lysate (Supplementary Fig. S4B). However, *Bip* mRNA was detected in lenses of all genotypes, but expression levels were similar between genotypes (Supplementary Fig. S4C). *Xbp1* mRNA splicing by IRE1, a sensor activated by ER stress, excises a 26-nucleotide long intron leading to production of XBP1 transcription factor.^60^ The levels of the spliced form of *Xbp1* mRNA were significantly different between lenses of the three genotypes of mice (*P* = 0.0003), and significantly higher in *Epha2^−^^/^^−^* compared to *Epha2^+/+^* lenses (*P* = 0.0006; Fig. 8A). However, its levels in *Epha2^+/^^−^* lenses were similar to those in *Epha2^+/+^* lenses. As *Epha2^+/^^−^* mice exhibited a delay in development of severe cataract, we hypothesized that levels of the spliced form of *Xbp1* mRNA may be increased in lenses of these mice at a later age. Thus, we further analyzed its levels in 14 to 19-week-old *Epha2^+/^^−^* and *Epha2^+/+^* lenses. The levels were found to be lower rather than higher in *Epha2^+/^^−^* compared to *Epha2^+/+^* lenses at that age (Fig. 8B). ATF6, another sensor of ER stress, upon translocation from the ER to golgi apparatus is proteolytically processed and the resulting processed protein, ATF6N, functions as a transcription factor.^60^ Expression of ATF6, predominantly the processed protein, was detected in lenses of all three genotypes of mice whereas both unprocessed and processed protein was detected in C2C12 cell lysate used as positive control (Supplementary Fig. S4B). The levels of the processed protein, ATF6N, were relatively higher (from ∼35 to 125%) in *Epha2^+/^^−^* and *Epha2^−^^/^^−^* than *Epha2^+/+^* lenses (see Supplementary Fig. S4B, Graph). Expression of CHOP, a pro-apoptotic protein activated by severe ER stress, was not detected in any genotype of lenses but, as expected, was detected in thapsigargin treated C2C12 cell lysate used as positive control (see Supplementary Fig. S4B). These data suggest that accumulation of the partial EPHA2-β-galactosidase fusion protein is accompanied by activation of a moderate ER stress and UPR in the lens. Notably, the fusion protein was detected in the soluble protein fraction extracted in RIPA buffer but not in the insoluble protein fraction of 18-week-old *Epha2^+/^^−^* and *Epha2^−^^/^^−^* lenses (see Supplementary Fig. S5A, Fig. 7, and data not shown) nor of 43-week-old *Epha2^+/^^−^* lenses (Supplementary Fig. S5B), suggesting that the mutant protein is present in lens cells but does not form insoluble aggregates.

**Figure 9.**
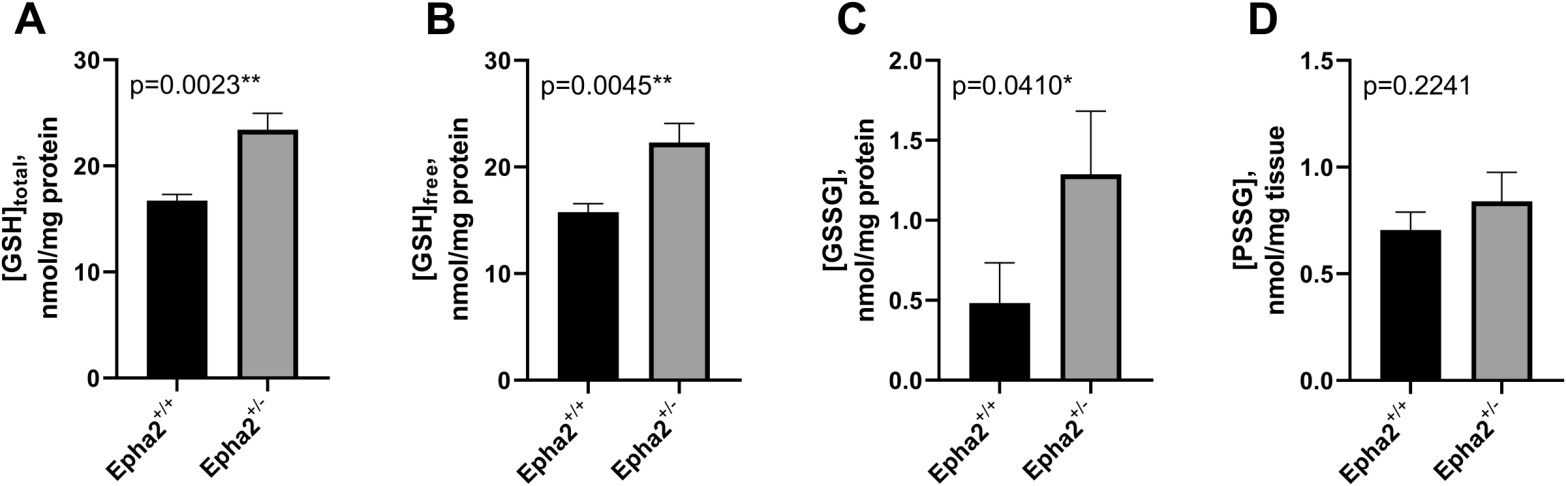
**Comparison of glutathione levels in *Epha2^+/+^* and *Epha2^+/^^−^* mouse lenses.** Concentrations of total soluble GSH (**A**), GSH in its free, reduced form (**B**), and oxidized forms as soluble GSSG (**C**) and protein-bound PSSG (**D**) were compared between 43-week-old *Epha2^+/^^−^* and age-matched *Epha2^+/+^* mouse lenses (*n* = 3), as described in Materials and Methods. Mean concentrations of total GSH, free GSH, and GSSG expressed as nmol/mg of lens protein and PSSG as nmol/mg dried tissue have been plotted. Two-tailed *t*-test *P* value of each comparison has been indicated. Significant *P* values are marked with *asterisks*; *P < 0.05, **P < 0.005. Error bars represent standard deviation from the mean.

Furthermore, to examine the effect of partial and complete loss of EPHA2 on redox state of the lens, we determined the levels of lenticular glutathione (GSH), the main antioxidant in the lens.^63^ Levels were evaluated for both, the free, reduced form, and oxidized forms, including glutathione disulfide (GSSG) and protein-bound glutathione (PSSG). Glutathione levels in lenses of 22-week-old *Epha2^−^^/^^−^* and 43-week-old *Epha2^+/^^−^* mice were compared to those in age-matched *Epha2^+/+^* mice. GSH/GSSG ratio, the metric useful for assessment of redox balance, was not used in this study because virtually undetectable levels of GSSG in several of the *Epha2^+/+^* lenses led to artifactually high ratios. Lenses of 43-week-old *Epha2^+/^^−^* mice had significantly higher concentrations of total GSH (*P* < 0.005), free GSH (*P* < 0.005), and GSSG (*P* < 0.05) compared to those of age-matched *Epha2^+/+^* mice (Figs. 9A–C), which indicated dysregulation of redox homeostasis in *Epha2^+/^^−^* lenses. However, concentrations of PSSG were not significantly different between lenses of the two genotypes. No significant difference in concentrations of total or free GSH, GSSG, or PSSG was noted between lenses of 22-week-old *Epha2^−^^/^^−^* and age-matched *Epha2^+/+^* mice likely due to small sample size (Supplementary Fig. S6). Taken together, these data suggest that partial and complete loss of functional EPHA2 affect the morphology and arrangement of lens fiber cells in an age-dependent manner and partial loss of the protein also affects glutathione redox balance of the lens, which contributes to cataract development in mice.

### Genetic Background Influences *Epha2-*Related Cataract Development

We hypothesized that the difference in timing of cataract progression in *Epha2^−^^/^^−^* mice found in our study and that reported by Jun et al.,^30^ and occurrence of cataract in *Epha2^+/^^−^* mice is due to difference in genetic backgrounds of *Epha2-*knockout mice used in the two studies. To test this hypothesis, we generated *Epha2-*knockout mice on mixed FVB:C57BL/6J (50:50) genetic background and determined cataract development in *Epha2^+/+^* (*n =* 17), *Epha2^+/−^* (*n =* 22), and *Epha2^−^^/^^−^* (*n =* 23) mice from 11 to 64 weeks of age or until severe cataract developed.

FVB/NJ mice, used for generating *Epha2-*knockout mice on mixed background, carry a spontaneous mutation in the *Bfsp2* gene^64^ that causes occasional anterior subcapsular and faint deeper lens opacities.^47^^,^^64^ To determine whether this mutation affects *Epha2-*related ACC development on mixed background, segregated analysis, by *Bfsp2* genotype (wild-type or heterozygous/homozygous mutant), was performed. *Epha2*-related cataract progression in *Epha2*^+/^*^−^* and *Epha2^−^^/^^−^* mice carrying homozygous or heterozygous mutant *Bfsp2*, was similar to those carrying the wild-type *Bfsp2* gene (see Supplementary Figs. S7, S8), suggesting that *Bfsp2* genotype did not affect *Epha2*-related cataract development in mice on mixed background. Thus, combined analysis of data from all three *Bfsp2* genotypes of mice is presented. The *Bfsp2* mutation reportedly also does not affect the cataract phenotype related to *Ephrin-A5* knockout.^41^

**Figure 10.**
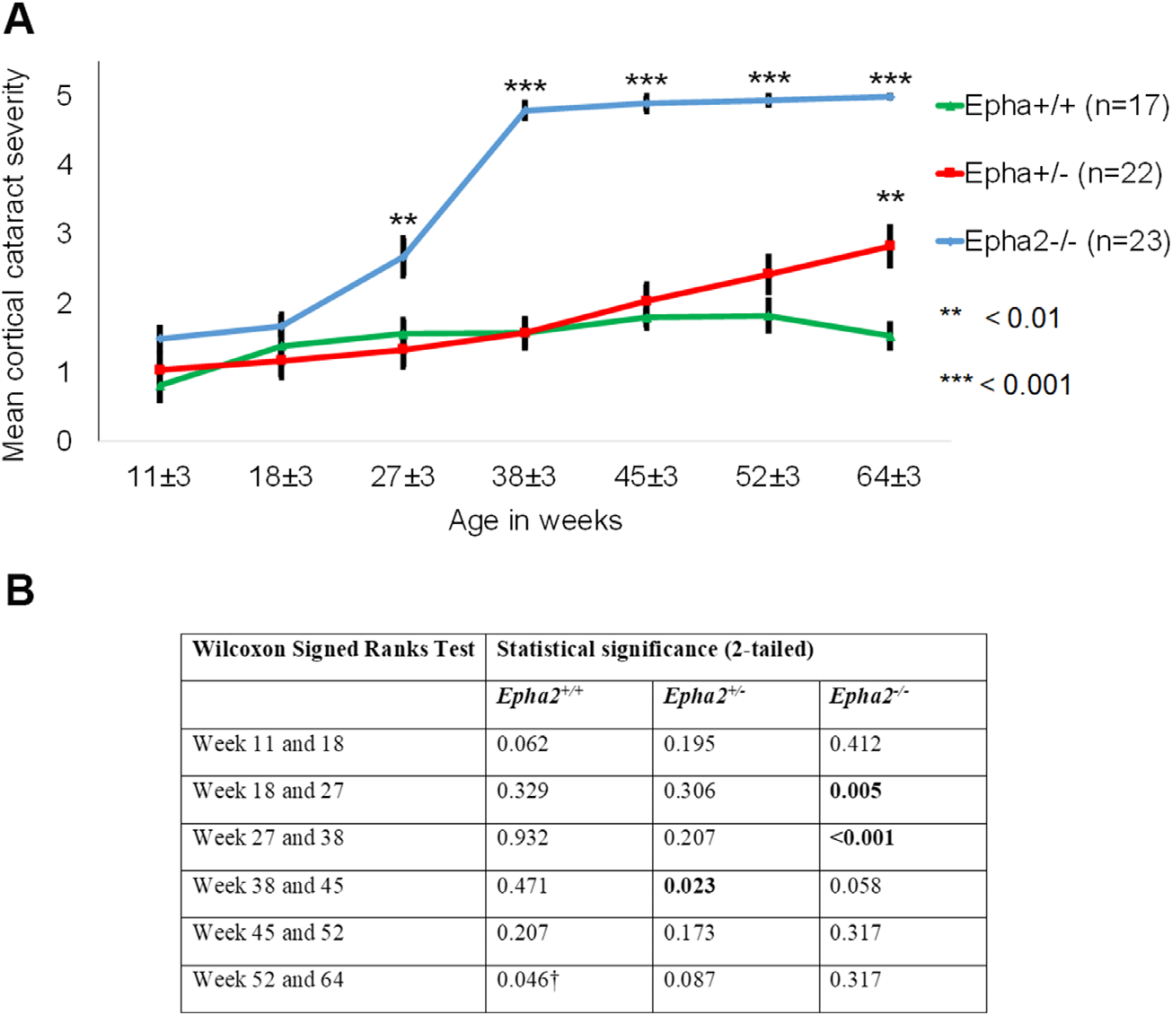
**Progression of anterior cortical cataract in *Epha2*-knockout mice on FVB:C57BL/6J mixed background.***Epha2^+/+^*, *Epha2^+/^^−^*, and *Epha2^−^^/^^−^* mice on FVB:C57BL/6J (50:50) mixed background were monitored for cataract development from 11 ± 3 weeks to 64 ± 3 weeks of age. (**A**) Mean cortical cataract grade in each genotype of mice at each time point is graphically represented. *Epha2^−^^/^^−^* mice developed severe cataract by 38 ± 3 weeks of age whereas *Epha2^+/^^−^* mice developed only moderate cataract by 64 ± 3 weeks of age. *Epha2^+/+^* mice presented with mild cataract up to 64 ± 3 weeks of age. The Mann Whitney *U* test *P* values from comparison with *Epha2^+/+^* mice are indicated. Error bars represent standard error of the mean. After an animal exhibited a cataract grade of 4 or 5, the same grade was attributed to subsequent time points for data analysis assuming no further increase in cataract severity. (**B**) The table shows Wilcoxon Signed Ranks test *P* values of comparison between time points indicating the effect of age on cataract progression in each genotype of mice. † Indicates likely artifact of anesthetic-induced cataract.

**Figure 11.**
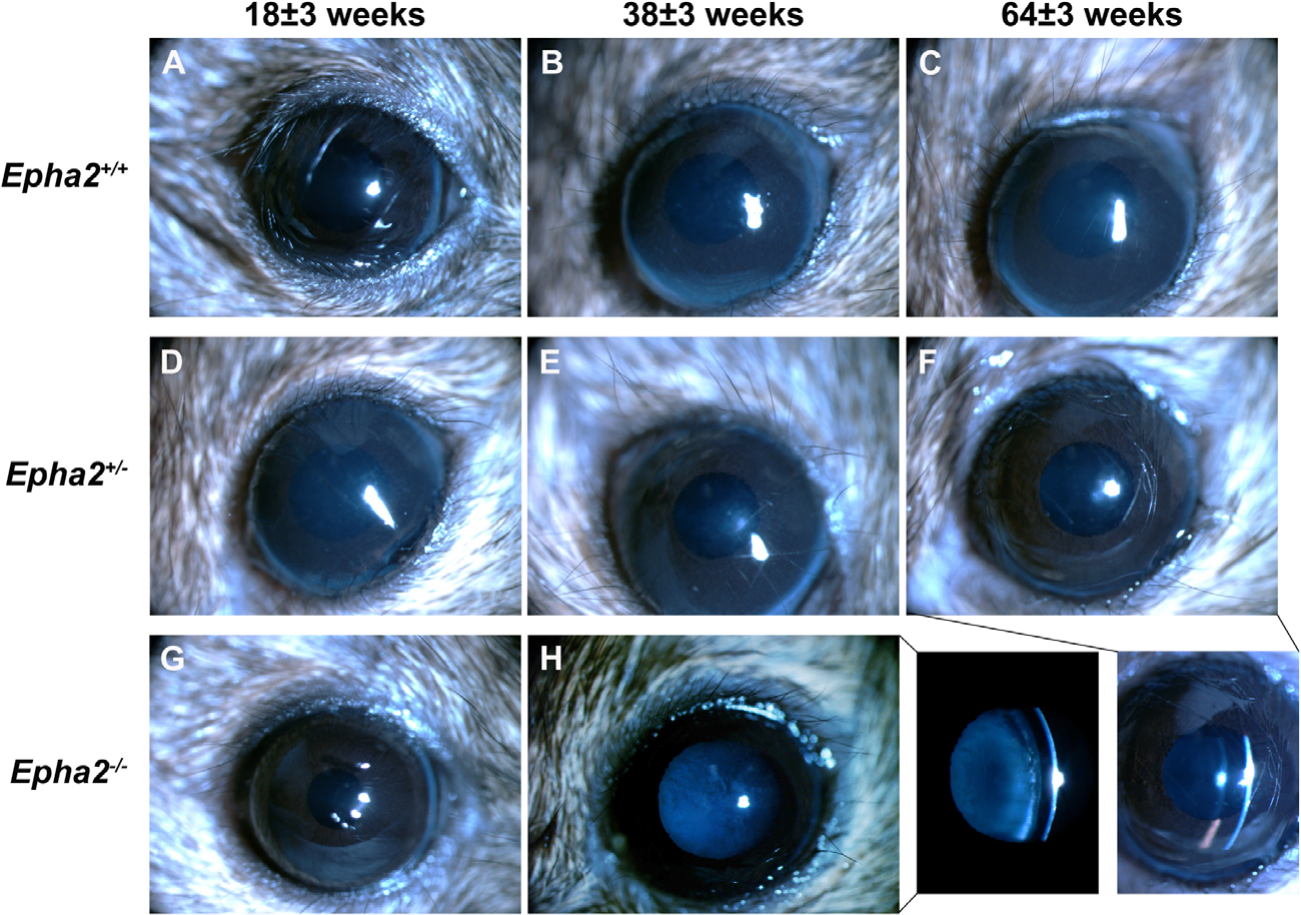
**Anterior cortical cataract developed in *Epha2-*knockout mice on FVB:C57BL/6J mixed background.** Representative ophthalmic images of *Epha2^+/+^* (**A, B,** **C**) and *Epha2^+/^^−^* (**D, E,** **F**) mice at 18 ± 3, 38 ± 3 and 64 ± 3 weeks of age and *Epha2^−^^/^^−^* (**G,** **H**) mice at 18 ± 3 and 38 ± 3 weeks of age, on FVB:C57BL/6J (50:50) mixed background, are shown. No cataract was evident in *Epha2*^+/+^, **C**, or in *Epha2*^+/−^, **F**, mice up to 64 weeks of age. *Epha2*^−/−^ mice progressively developed severe ACC by 38 ± 3 weeks of age, **H**. The bottom right panel shows cross-section illumination images of the *Epha2^−^^/^^−^* and *Epha2^+/^^−^* eyes seen in panels **H** and **F**, respectively. Magnification, times 40.

In *Epha2^+/+^* mice on mixed background, only mild ACC (grade < 2) was observed up to 64 weeks of age (Figs. 10, 11, Supplementary Table S3) and attributed to anesthesia. *Epha2^+/^^−^* mice exhibited mild ACC up to 45 weeks of age and moderate ACC (grade >2) by 64 weeks of age compared to age-matched *Epha2^+/+^* mice (*P* < 0.01; see Figs. 10A, 11, Supplementary Table S3). *Epha2^−^^/^^−^* mice, compared to *Epha2^+/+^* mice, displayed mild ACC by 18 weeks that progressed to moderately severe ACC by 27 weeks (grade >2, *P* < 0.01) and severe ACC by 38 weeks of age (grade 4 or 5, *P* < 0.001; see Figs. 10A, 11, Supplementary Table S3). Increase in cataract severity between 18 and 27 weeks and 27 and 38 weeks of age in this genotype was significant (Fig. 10B).

**Figure 12.**
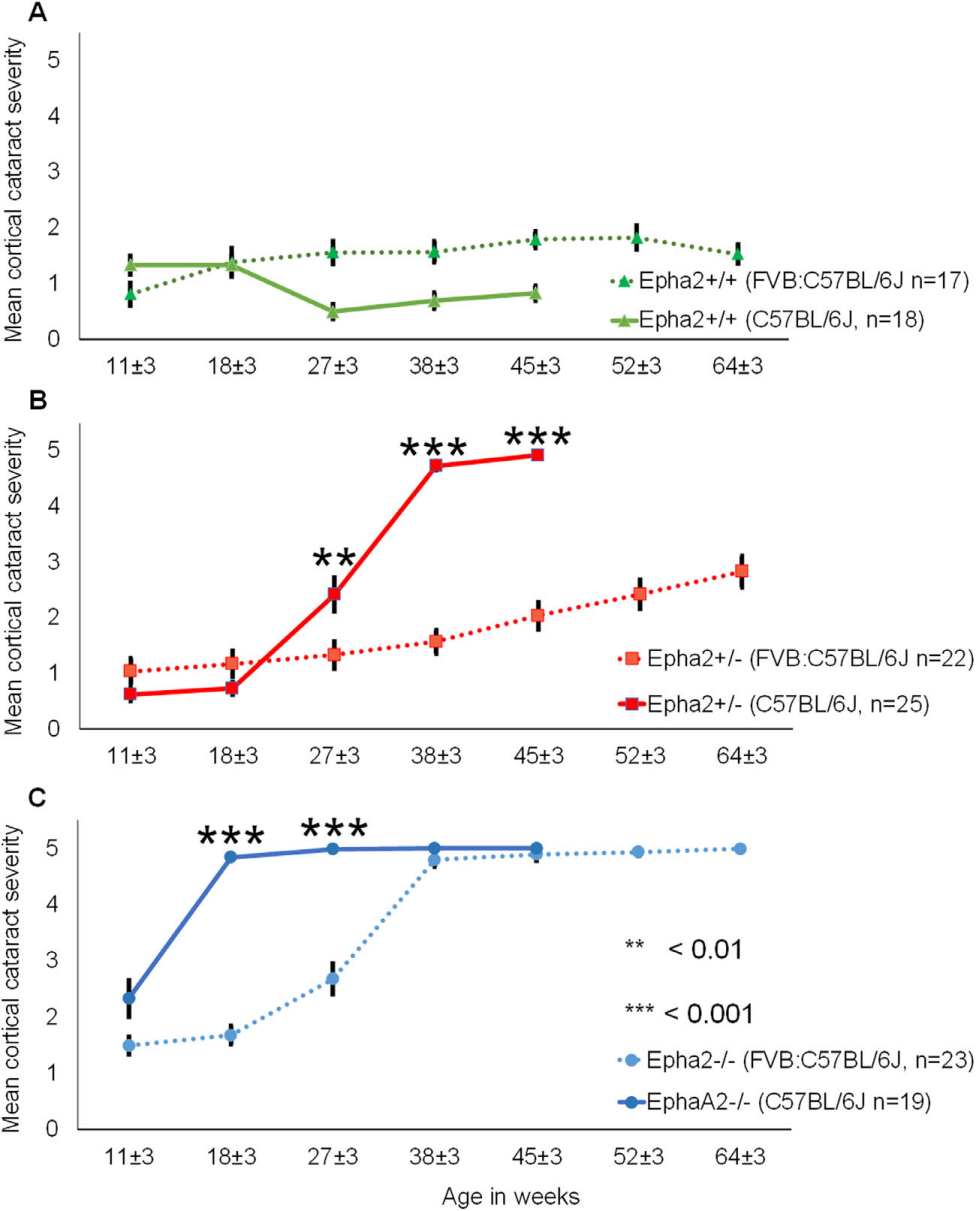
**Comparison of progression of anterior cortical cataract in *Epha2*-knockout mice on C57BL/6J and FVB:C57BL/6J mixed backgrounds.** Anterior cortical cataract severity was compared between C57BL/6J (*solid lines*) and FVB:C57BL/6J (50:50) mixed (*dotted lines*) backgrounds in *Epha2^+/+^* (**A**), *Epha2^+/^^−^* (**B**), and *Epha2^−^^/^^−^* (**C**) mice, over time. Group sizes are indicated in each panel. Mean ACC grade in a genotype of mice on each background at each time point has been plotted. On both the genetic backgrounds, *Epha2^+/+^* mice developed only mild cataracts up to 45 weeks of age that persisted in mice on the mixed background up to 64 ± 3 weeks of age, **A**. *Epha2^+/^^−^* mice on C57BL/6J background developed moderate cataracts by 27 weeks of age (*P* < 0.01) and severe cataracts by 38 or 45 weeks of age (*P* < 0.001) compared to mild cataracts in age-matched *Epha2^+/^^−^* mice on the mixed background; *Epha2^+/^^−^* mice on mixed background developed only moderate cataract up to 64 ± 3 weeks of age, **B**. *Epha2^−^^/^^−^* mice on C57BL/6J background developed severe cataracts by 18 ± 3 or 27 ± 3 weeks of age whereas those on the mixed background developed severe cataract by 38 ± 3 weeks of age, **C**. The latter mice had significantly less severe cataracts at 18 and 27 weeks of ages (*P* < 0.001). The *p* values from the Mann Whitney *U* test are indicated. Error bars represent standard error of the mean.

Next, we compared the effect of genetic background on cataract progression in each of the three genotypes of mice. *Epha2^+/+^* mice on both genetic backgrounds exhibited only mild anesthetic-induced cataract at all time points (Fig. 12A); although statistically significant (*P* < 0.001), the difference is unlikely to be biologically relevant. On C57BL/6J background, *Epha2^+/^^−^* mice developed moderate cataract at 27 weeks (*P* < 0.01) and severe cataract by 38 and 45 weeks of age (*P* < 0.001; Fig. 12B); however, the mice on mixed background developed only moderately severe cataracts by 64 weeks of age. Similarly, *Epha2^−^^/^^−^* mice on C57BL/6J background developed significantly severe cataract by 18 and 27 weeks of age compared to those on mixed background (*P* < 0.001; Fig. 12C) that developed severe cataract much later, by 38 weeks of age. Together, these results suggest that *Epha2*-related cataract develops significantly faster on C57BL/6J than on FVB:C57BL/6J (50:50) mixed background. This also explains the reason for cataract development in *Epha2^+/^^−^* mice on C57BL/6J background discovered in this study.

### Sex Influences *Epha2*-Related Cataract Progression

Female subjects are at a higher risk of developing age-related cataract and some epidemiological studies suggest a protective effect of hormone replacement therapy in post-menopausal women.^8^^,^^65^ Moreover, estrogen reportedly affects EPHA2 signaling.^44^ Thus, we hypothesized that sex may influence *Epha2*-related cataract development. To determine this, we compared cataract grades between male and female *Epha2^+/^^−^* and *Epha2^−^^/^^−^* mice on the two genetic backgrounds.

**Figure 13.**
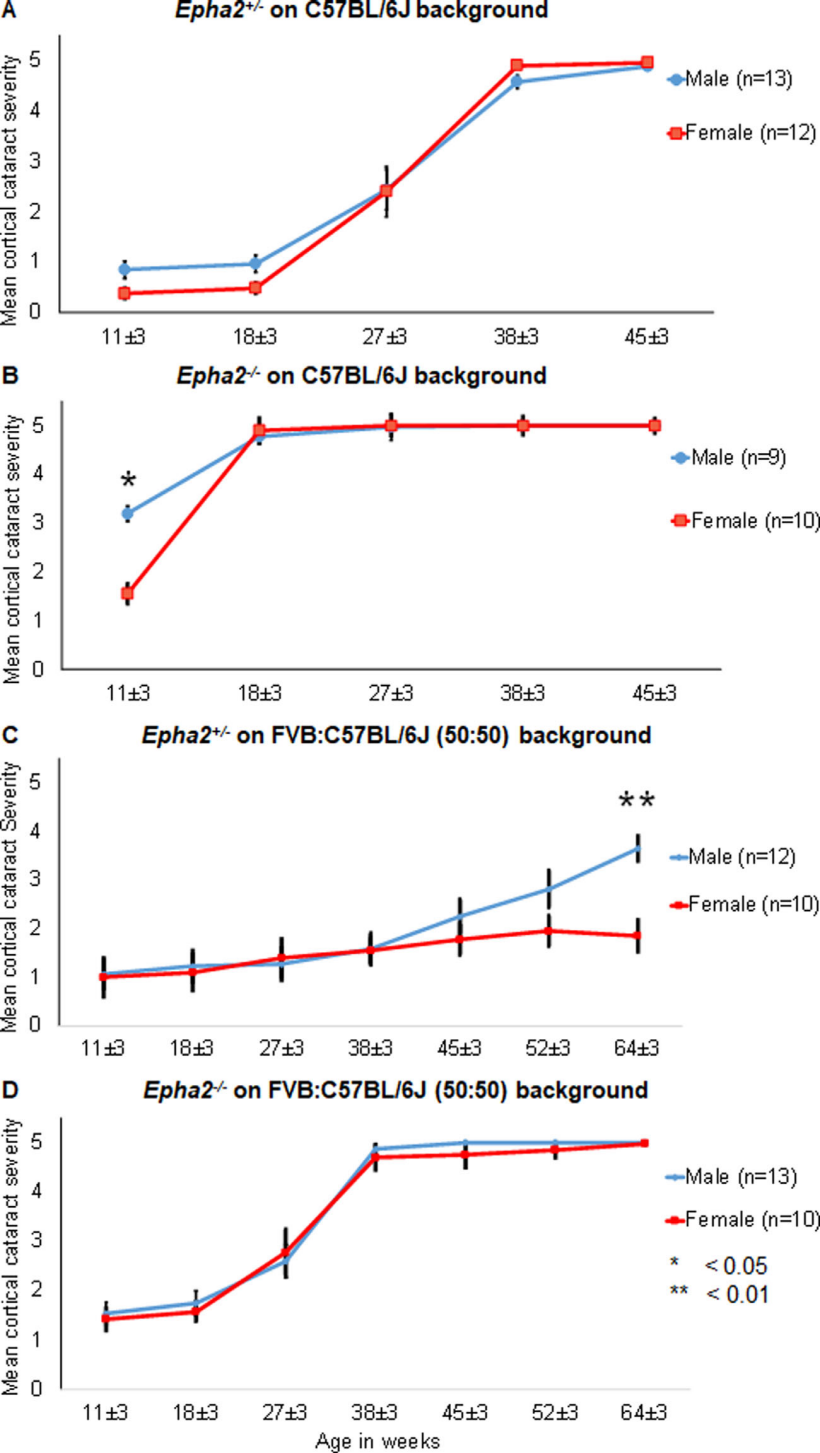
**Comparison of progression of anterior cortical cataract between male and female *Epha2*-knockout mice on C57BL/6J and FVB:C57BL/6J mixed backgrounds.** Anterior cortical cataract severity was compared between male (*blue lines*) and female (*red lines*) *Epha2^+/^^−^* and *Epha2^−^^/^^−^* mice on C57BL/6J and FVB:C57BL/6J (50:50) mixed genetic backgrounds. (**A**) Male and female *Epha2^+/^^−^* mice on C57BL/6J background showed similar progression of cataract from 11 ± 3 to 45 ± 3 weeks of age. (**B**) Female *Epha2^−^^/^^−^* mice on C57BL/6J background displayed milder cataract compared to male mice at 11 ± 3 weeks of age (*P* < 0.05) whereas both male and female mice exhibited similar severe cataracts at 18 ± 3 weeks of age that persisted subsequently. (**C**) Both male and female *Epha2^+/^^−^* mice on the mixed genetic background displayed mild cataract up to 45 ± 3 weeks of age. Male mice gradually developed moderately severe cataract by 64 ± 3 weeks of age whereas age-matched female mice exhibited significantly milder cataract up to that age (*P* < 0.01). (**D**) Male and female *Epha2^−^^/^^−^* mice on the mixed background exhibited similar rate of progression of cataract from 11 ± 3 to 64 ± 3 weeks of age. The *P* values from the Mann Whitney *U* test are indicated. Error bars represent standard error of the mean.

There was no difference in cataract severity between *Epha2^+/^^−^* male (*n =* 13) and female (*n =* 12) mice from 11 to 45 weeks of age, on C57BL/6J background (Fig. 13A). Whereas *Epha2^−^^/^^−^* male mice (*n =* 9) on C57BL/6J background displayed significantly higher cataract severity than female mice (*n* *=* 10) at 11 weeks of age (*P* < 0.05); both male and female mice progressed to develop severe cataract by 18 weeks of age (Fig. 13B).

On mixed genetic background, both male (*n =* 12) and female (*n =* 10) *Epha2^+/^^−^* mice exhibited mild cataract up to 38 weeks of age, thereafter, male mice displayed higher grade cataracts than female mice and significantly higher at 64 weeks of age (*P* < 0.01; Fig. 13C). In contrast, there was no difference in cataract severity between male (*n =* 13) and female (*n =* 10) *Epha2^−^^/^^−^* mice on mixed background at any age (Fig. 13D). Overall, these results indicate that sex has a small effect on rate of progression of *Epha2*-related cataract and male mice start to develop cataract earlier than female mice.

## Discussion

Involvement of the *EPHA2* gene in cataract development in both humans and mice indicates a crucial role of EPHA2 signaling in the lens. In the present study, using an *Epha2-*knockout mouse model, we investigated the effect of various biological modifiers, *Epha2* genotype, age, sex, and genetic background, on *Epha2*-related cataract development. In contrast to previous studies, this study shows that partial deficiency of functional *Epha2* gene in mice also leads to ACC albeit at a later age than complete deficiency. Moreover, it shows the effect of genetic background and sex on progression of *Epha2*-related cataract.

The anterior cortical lens opacity observed in this study is similar to that reported by Jun et al. in null mice of this strain and another strain carrying a different *Epha2*-knockout mutation introduced using secretory gene trapping strategy.^30^ However, homozygous *Epha2*-knockout mice carrying an insertion mutation in the *Epha2* gene and predicted to produce truncated protein (Jackson Laboratory strain #006028) generated by homologous recombination,^66^ on C57BL/6J background, develop mild nuclear cataract and exhibit smaller lenses with impaired refractive quality and suture formation; reports regarding development of age-related cataract in these mice are conflicting.^38^^,^^39^^,^^67^ The difference in lens phenotype between strains generated by secretory gene-trap approach and homologous recombination is likely due to differences in *Epha2*-knockout mutations and protein products produced, and is similar to differences in cataract phenotype observed in humans with different genetic mutations in *EPHA2*.^33^^,^^34^ Additionally, because in strain #006028 the mutant protein is absent in the lens^38^ initiation of compensatory mechanisms involving another EPH receptor may explain the subtle phenotype. However, as shown by our data, in the secretory gene-trap strains the mutant fusion-protein persists in the lens. This may prevent initiation of a compensatory mechanism or have a dominant negative effect, leading to a more severe phenotype. Further research is warranted to investigate these possibilities.

This study shows that disruption of shape, size, and packing of lens fiber cells underlies ACC development in the *Epha2*-knockout model used here and suggests that loss of functional EPHA2 receptor from the cell membrane is likely the primary cause of this cellular disorganization. Our data show that the partial EPHA2-β-galactosidase fusion protein produced in this strain accumulates in lens cells and is accompanied by induction of a moderate unfolded-protein response indicating some ER stress. Unfolded-protein response is activated through three stress sensing pathways - IRE1, ATF6, and PERK.^60^ Acute or mild ER stress triggers a cytoprotective UPR for restoring ER homeostasis by reducing un/misfolded protein burden and/or facilitating protein folding whereas chronic or severe ER stress leads to a cytotoxic response that results in cell death.^60^ Activation of XBP1/ IRE1 and ATF6 pathways in *Epha2* null and ATF6 pathway in *Epha2^+/^^−^* mice in the absence of CHOP activation that leads to cell apoptosis suggests that the response is most probably cytoprotective and is induced for maintaining ER homeostasis. Consonant with this argument, the accumulated protein is detected in the soluble and not insoluble protein fraction of *Epha2*-knockout lenses. Hence, its aggregation or mis-folding is unlikely to underlie cataractogenesis in this model. The presence of a functional β-galactosidase reporter protein in this model^30^ also refutes aggregation of the fusion protein. Overexpression of unphosphorylated HSP25, the oligomeric form that acts as a chaperone, reported in lenses of *Epha2* null mice^30^ may facilitate solubility of the fusion protein. Alternatively, it may be related to aging as HSP25 overexpression was also found in adult wild-type lenses, although to a lesser extent.^30^ In this study, lenses of *Epha2^+/^^−^* mice, despite accumulation of the fusion protein in cells, remained clear when wild-type EPHA2 protein produced by the normal allele was localized to the cell membrane (at 18 weeks of age), and developed severe cortical cataracts when it was lost from the cell membrane (at 45 weeks of age). Downregulation of *Epha2* expression in the lens with age^30^ may explain the loss of wild-type protein from the cell membrane in older *Epha2^+/^^−^* lenses. Hence, loss of functional EPHA2 protein is the most likely cause of cataractogenesis in this model. However, the possibility that intracellular accumulation of the fusion protein contributes to scattering of light cannot be discounted.

Our data show an increase in oxidized glutathione (GSSG) in lenses of *Epha2^+/^^−^* mice at the age when they develop severe cortical cataracts, suggesting an increase in reactive oxygen and/or nitrogen species in these lenses. However, lenses of these mice also exhibited significantly higher levels of reduced (free) GSH than those of matched wild-type mice. GSH levels in the lens are maintained via a combination of de novo synthesis from constituent amino acids, recycling, and uptake from surrounding tissues.^68^ There is precedent for upregulation of one or more of these pathways as a protective response to oxidative stress^69^ that could explain the increased GSH levels in *Epha2^+/^^−^* lenses. Further research is required to elucidate the role of GSH and oxidative stress in development of cataract due to loss of functional EPHA2.

We found a temporal effect of *Epha2* genotypes on development of cortical cataract in mice on two genetic backgrounds, an inbred and a mixed background. This demonstrates that age as a function of genotype has a significant effect on *Epha2*-related cataract development, which correlates with association of variants in the human *EPHA2* gene with the risk of developing age-related cataract.^29^^,^^30^^,^^32^^,^^70^ The genetic backgrounds also conferred a temporal rather than a phenotypic effect on *Epha2*-related cataract. Genetic background is known to influence disease phenotype and severity in genetically and nongenetically induced animal models of diseases, for example, in animal models of epilepsy^71^ and cataract.^40^^,^^41^^,^^72^^,^^73^ An earlier development of cortical cataract in *Epha2^−^^/^^−^* and *Epha2^+/^^−^* mice on C57BL/6J background than on FVB:C57BL/6J (50:50) mixed background is most likely due to genetic modifiers and indicates the presence of susceptibility factors in the former and/or resistance factors in FVB/NJ background. Similarly, racial differences in susceptibility to cataract have been found in humans.^74^^,^^75^ Identification of the genetic modifiers in C57BL/6J and FVB/NJ backgrounds, may reveal additional risk factors for age-related cataract. An effect of genetic background on *Epha2*-related cataract in mice is consistent with differences in incidence, severity, and penetrance of cataract among individuals carrying mutations in the *EPHA2* gene.^33^^,^^76^

Sex was found to have a significant, albeit small, effect on rate of progression of *Epha2*-related cataract in mice. *Epha2^−^^/^^−^* female mice on C57BL/6J background were protected from cataract at a younger age but progressed to develop severe cataract like male mice at a later age. A similar protective effect was seen in *Epha2^+/^^−^* female mice on mixed background. A protective effect was not evident in *Epha2^+/^^−^* female mice on C57BL/6J background and *Epha2^−^^/^^−^* female mice on mixed background, probably due to small sample size and intermediate rate of progression of cataract in these mice. Nevertheless, the effect of sex on cataract progression in mice is consistent with differences in prevalence of age-related cataract between male and female subjects reported in some epidemiological studies.^2^^,^^8^ Differences in estrogen levels between female and male mice likely underlie the protective effect of female gender during early stages of cataract development in *Epha2*-knockout mice and correlates with reduced susceptibility to age-related cataract in women on hormone replacement therapy.^65^ Further research is warranted to understand the role of estrogen in *Epha2*-related cataract.

In summary, this study, we believe for the first time, demonstrates that partial deficiency of the *Epha2* gene is sufficient to cause cataract and shows an age-dependent effect of partial and complete deficiency of EPHA2 on cataract development in mice. It shows that female gender confers some protection and delays the onset, and genetic background has a major effect on rate of progression of *Epha2-*related cataract in mice. These findings reiterate that cataract is a multifactorial disease and suggest that interaction between genetic and other biological factors likely determines susceptibility to *EPHA2*-related cataract in humans.

## Supplementary Material

Supplement 1
