# Supplementary material for: Genotype, Age, Genetic Background, and Sex Influence Epha2-Related Cataract Development in Mice
Source: Invest Ophthalmol Vis Sci. 2021 Sep 8;62(12):3. doi: 10.1167/iovs.62.12.3 (PMC8431977; doi:10.1167/iovs.62.12.3)
Supplement: Supplement 1 [file iovs-62-12-3_s001.pdf]

Supplementary material

Methods

Lens homogenization

Lenses were homogenized in a serine-borate buffer (SBB) consisting of 100 mM Tris-HCl (MilliporeSigma, MA, USA), 5 mM L-serine (MilliporeSigma, MA, USA), 10 mM boric acid (Fisher Scientific, MA, USA), and 1 mM diethylenetriaminepentaacetic acid (MilliporeSigma, MA, USA) prepared in ultra-pure water and titrated to a pH of 7.0 with NaOH. Each lens was placed into a separate RINO™ (Next Advance, Inc., NY, USA) microcentrifuge tube containing a single UFO homogenization bead (Next Advance, Inc., NY, USA). To each tube 30 µL of 100 µM N-acetylcysteine (internal standard) and 300 µL of chilled SBB were added. All samples and standards were handled identically and homogenized in tandem using a Bullet Blender Storm (Next Advance, Inc., NY, USA) set at maximum speed for two cycles of 5 min. The homogenizer was operated in a freezer to prevent the samples from heating. Beads were promptly removed after homogenization using a rare-earth magnet. Samples were then centrifuged at 5000 × g for 5 min at 4 °C to remove cellular debris.

Calibration standards

Reduced glutathione (MilliporeSigma, MA, USA) was used to prepare the calibration curves for determination of free and total soluble GSH, and N-acetylcysteine (MilliporeSigma, MA, USA) was used as an internal standard. Glutathione disulfide (MilliporeSigma, MA, USA) was used to prepare the calibration curve for estimation of PSSG. All calibration standards were prepared in parallel with the lens samples and were subjected to identical storage and handling procedures.

Estimation of protein-bound glutathione

Sample preparation

To precipitate the protein, 200 μL aliquots of the lens homogenates were mixed with ice-cold 80% methanol in pre-weighed microcentrifuge tubes. The samples were centrifuged at 7400 ×g for 15 min at 4°C. The supernatants containing unbound GSH and GSSG were removed, and the protein pellets were washed twice with ice-cold 80% methanol. The protein pellets were dried in a Vacufuge 5301 (Eppendorf, NY, USA) at room temperature for 4.5 hours. The weight of each tube and pellet was recorded. Each pellet was then resuspended in 0.5 mL of a pH 7.0, 100 mM ammonium bicarbonate / formic acid buffer. The samples were then reduced by mixing 350 μL aliquots of the suspended protein samples with 10 μL of 75 mM dithiothreitol (DTT) and 140 μL of LC-MS grade water. The protein samples were shaken vigorously for one hour to allow for reduction and liberation of protein-bound glutathione. The samples were then centrifuged at 11000 ×g for 15 min at room temperature. Finally, 9 μL of supernatant was diluted with 900 μL of 0.1% formic acid (LC-MS grade) and then filtered through a 0.22 μm nylon membrane filter.

Chromatography

A Shimadzu Prominence UFLC system consisting of a degasser (DGU-30A3), two pumps (LC-20 AD XR), an autosampler (SIL-20AC XR), and a column oven (CTO-20A), were coupled to an AB Sciex 4000QTRAP LC/MS/MS system and controlled via AB Sciex Analyst Software to carry out the separation and quantification of analytes in this study. A Phenomenex HydroRP column (4 μm, 250 × 2 mm) was employed for separation under positive electrospray ionization (ESI) conditions. UFLC was performed at 40°C with binary flow at a rate of 0.3 mL/min with mobile phase A consisting of 0.1% formic acid in ultra-pure water and mobile phase B consisting of 0.1%

formic acid in acetonitrile. The method began with a pre-run equilibration of 8 minutes at 100% A, followed by injection of 20 μL of sample. A linear gradient increased to 100% B from 0-8 minutes, isocratic 100% B from 8-10 minutes, and finally a linear decrease to 100% A from 10-13 minutes. GSH was detected in multiple reaction monitoring (MRM) mode with Q1 m/z 307.985 and Q3 m/z 179.100.

Calibration curves

Determination of free GSH, GSSG, and total soluble GSH

The GSH standard curve (Supplementary Figure S1) was constructed by plotting the known concentrations of GSH calibration standards versus the corresponding peak areas. The internal standard was employed to account for minor variations in sample volume due to evaporation since the same amount of NAC should be present in each sample. Correction of GSH peak area was done by multiplying each individual GSH peak area ($A_{GSH,i}$) by the ratio of the average NAC peak area ($\overline{A}_{NAC}$) to the NAC peak area of the individual sample ($A_{NAC,i}$) as follows:

$$A_{GSH,i}\ (corrected) = \frac{\overline{A}_{NAC}}{A_{NAC,i}} \times A_{GSH,i}$$

In order to validate the accuracy of the GSH standard curve for determination of GSSG following reduction, reduced standards prepared with known GSSG concentrations were plotted against the resulting peak areas to confirm a direct correlation with the GSH calibration curve (Supplementary Figure S1). GSSG was calculated by subtracting free GSH from total GSH and dividing by two. All concentrations are expressed in nanomoles per milligram of protein as determined from the Bradford assay. The peak areas resulting from reduction of GSSG check standards matched the

GSH calibration curve, indicating that complete reduction of GSSG and stoichiometric yield of GSH were achieved over the full concentration range of interest.

Estimation of PSSG

Levels of PSSG in the lens samples were estimated by solving the regression equation fit to a calibration curve (Supplementary Figure S2) generated from analyzing GSSG standards of known concentration. Standards of GSSG were used since analytical-grade PSSG is not commercially available. The peak areas measured correspond to GSH that was liberated from protein by reduction with DTT. The concentration of PSSG was expressed as nmol/mg of dried tissue.

Supplementary Table 1: Comparison of anterior cortical cataract severity in different age groups of *Epha2*$^{+/+}$ and *Epha2*$^{+/-}$ mice on C57BL/6J background. *p* values of Mann Whitney *U* test are indicated. Significant values are in bold font. SEM= Standard error of the Mean.

	**Mean cataract grade± SEM**		
**Age in weeks**	***Epha2*$^{+/+}$**	***Epha2*$^{+/-}$**	***p* value**
**11±3**	1.33±0.33	1.50±0.76	0.817
**18±3**	1.50±0.17	1.03±0.19	0.082
**27±3**	1.67±0.20	2.14±0.26	0.259
**38±3**	0.69±0.16	4.44±0.18	**<0.001**
**45±3**	1.20±0.20	5.00±0.00	**0.001**
**52±3**	0.83±0.46	4.57±0.17	**0.002**

Supplementary Table 2: Mean anterior cortical cataract severity in *Epha2*$^{+/+}$, *Epha2*$^{+/-}$ and *Epha2*$^{-/-}$ mice on C57BL/6J background from 11 to 45 weeks of age. SEM= Standard error of the Mean.

	**Mean grade±SEM**		
**Age in weeks**	***Epha2*$^{+/+}$**	***Epha2*$^{+/-}$**	***Epha2*$^{-/-}$**
**11±3**	1.33±0.16	0.62±0.11	2.33±0.32
**18±3**	1.33±0.16	0.73±0.11	4.84±0.07
**27±3**	0.50±0.13	2.42±0.31	4.99±0.01
**38±3**	0.69±0.14	4.73±0.08	5.0±0.0
**45±3**	0.83±0.13	4.92±0.04	5.0±0.0

Supplementary Table 3: Mean anterior cortical cataract severity in *Epha2*$^{+/+}$, *Epha2*$^{+/-}$ and *Epha2*$^{-/-}$ mice on C57BL/6J:FVB(50:50) mixed background from 11 to 64 weeks of age. SEM= Standard error of the Mean.

	**Mean grade±SEM**		
**Age in weeks**	***Epha2*$^{+/+}$**	***Epha2*$^{+/-}$**	***Epha2*$^{-/-}$**
**11±3**	0.81±0.21	1.03±0.24	1.49±0.16
**18±3**	1.38±0.26	1.17±0.24	1.67±0.16
**27±3**	1.56±0.21	1.33±0.25	2.67±0.27
**38±3**	1.57±0.19	1.57±0.21	4.79±0.11
**45±3**	1.79±0.15	2.03±0.24	4.89±0.11
**52±3**	1.82±0.22	2.42±0.26	4.93±0.06

**64±3**	1.53±0.17	2.83±0.28	4.99±0.01

Supplementary Figure S1: Calibration curve for determination of free GSH, GSSG, and total soluble GSH.

Standards of known concentrations of GSH and GSSG were reduced and processed as described in Supplementary Methods. The yellow diamonds represent GSH calibration standards (0-50 μM). The black line is a linear least-squares regression fit to data from GSH standards, and the blue squares represent GSSG quality-check standards (0-25 μM). Concentrations of GSSG were multiplied by 2 since the stoichiometric yield of GSSG reduction is 2 GSH per GSSG.

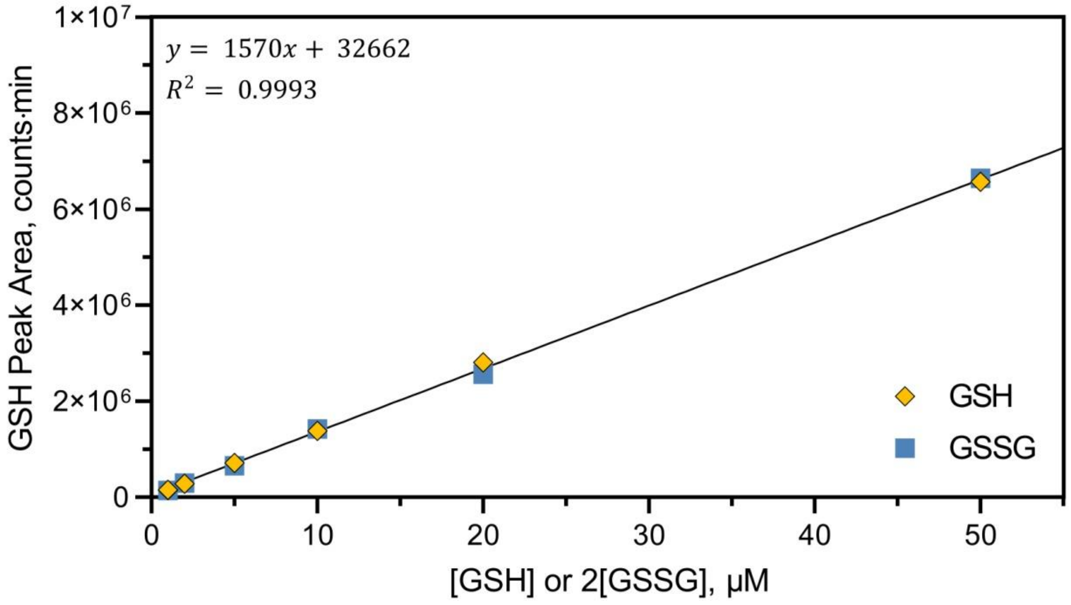

Supplementary Figure S2: Calibration curve for estimation of [PSSG].

Glutathione disulfide (GSSG) standards of known concentration were reduced with DTT and processed in parallel with lens samples. Plotted are the known concentrations (multiplied by a factor of two) vs. the resulting chromatographic peak areas corresponding to GSH. The yellow diamonds represent peak areas resulting from reduced GSSG standards. The black line is a linear least-squares regression fit to the data. Concentrations of GSSG were multiplied by 2 since the stoichiometric yield of GSSG reduction is 2 GSH per GSSG while reduction of PSSG yields 1 GSH per PSSG.

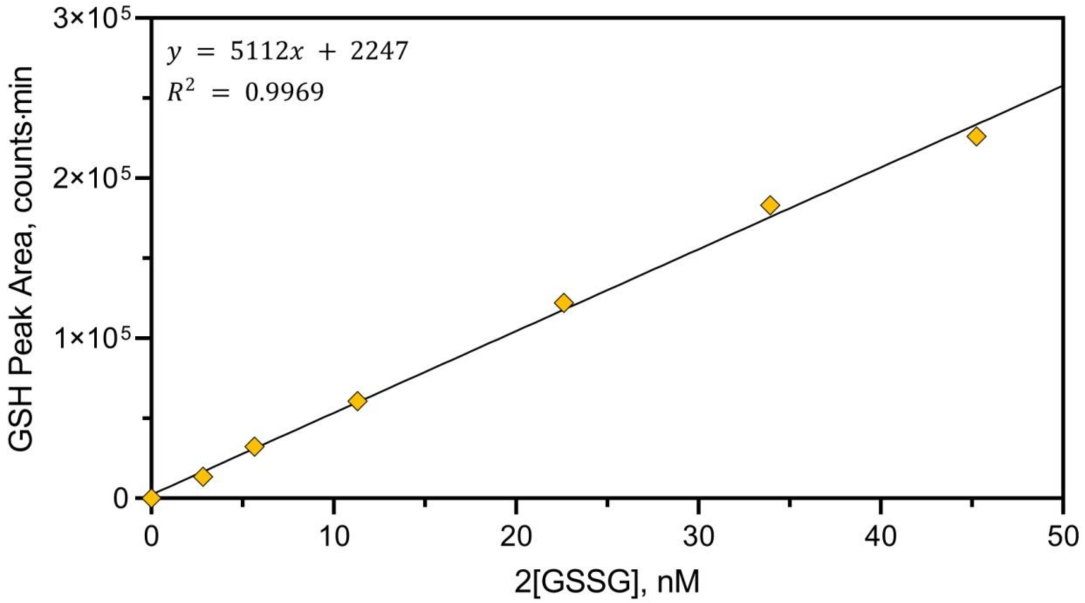

Supplementary Figure S3: Accumulation of mutant EPHA2-β-galactosidase fusion protein in *Epha2*-knockout mouse lenses detected by immunolabeling.

Representative confocal microscopy images of lens sections of 18-week-old *Epha2*$^{+/+}$ (A), *Epha2*$^{+/-}$ (B) and *Epha2*$^{-/-}$ (C) and 45-week-old *Epha2*$^{+/+}$(D) and *Epha2*$^{+/-}$ (E) mice on C57BL/6J background immunolabeled with anti-β-galactosidase antibody (red) and DAPI (blue) for staining the nuclei. Lenses of 18 (B) and 45-week-old *Epha2*$^{+/-}$ (E) and 18-week-old *Epha2*$^{-/-}$ (C) mice showed the presence of granular β-galactosidase protein in cells. The positive labeling likely indicates accumulation of the partial EPHA2-β-galactosidase fusion protein in lens cells. Similar labeling was absent in lenses of 18 (A) and 45-week-old *Epha2*$^{+/+}$ (D) mice. Sections incubated with rabbit IgG served as negative control and showed minimal non-specific labelling (F). Scale-bars 20µm.

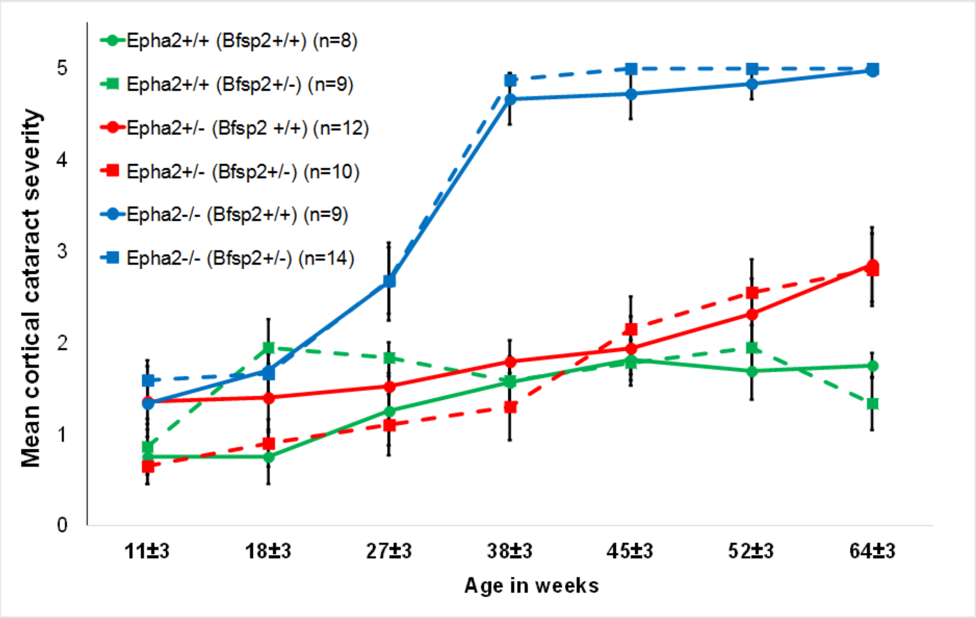

Supplementary Figure 3

Supplementary Figure S4: Assessment of activation of unfolded-protein response in *Epha2*-knockout mouse lenses.

A. Total soluble proteins extracted from lenses (n=2) of 18±1, 27±1, and 44±1 week-old *Epha2*$^{+/+}$, *Epha2*$^{+/-}$ and *Epha2*$^{-/-}$ mice on C57BL/6J background were analysed by western blotting using anti-BiP antibody. BiP expression was not detected in lenses of any genotype of mice at any age (top panel) indicating no obvious activation of unfolded protein response (UPR). Expression of BiP in HeLa cell lysate used as positive control demonstrated antibody specificity; the protein migrated slightly faster than its expected size of 78 kDa. **B.** Total soluble proteins extracted from lenses (n=2) of 9 or 10 week-old *Epha2*$^{+/+}$ and 8-week-old *Epha2*$^{+/-}$ and *Epha2*$^{-/-}$ mice on C57BL/6J background were analysed by western blotting using anti-BiP, anti-ATF6 and anti-CHOP antibody as indicated. BiP (top panel) and CHOP (third panel) expression was not detected in lenses of any genotype of mice indicating no obvious activation of UPR and cell apoptosis, respectively. Expression of CHOP in thapsigargin treated and not in untreated C2C12 cell lysates respectively used as positive and negative control demonstrated antibody specificity. ATF6 expression was detected in lenses of all genotypes of mice (second panel); expression of the processed protein (bracket, ATF6N) predominated that of unprocessed protein (arrow). Normalized ATF6N levels were ~35 to 125% higher in *Epha2*$^{+/-}$ and *Epha2*$^{-/-}$ relative to *Epha2*$^{+/+}$ lenses (graph) indicating activation of UPR; expression was normalized to that of crystallins (bottom panel); an experimental artifact precluded quantification in a *Epha2*$^{+/+}$ and a *Epha2*$^{+/-}$ lens lysate (2nd and 3rd lane, respectively). Expression of unprocessed and processed ATF6 in HeLa and C2C12 cell lysates demonstrated antibody specificity; the protein forms migrated somewhat differently in HeLa and C2C12 cell lysates likely due to species related differences; difference in protein profile between mouse lens and mouse C2C12 myoblast cell lysates is likely due to cell

type dependent differences in processing. Protein bands corresponding to lens crystallins were visible in all lens lysates and served as internal loading control (bottom panels in A and B). M, molecular weight of protein standards in kiloDaltons. Red indicates signal saturation. **C.** *Bip* mRNA was amplified from lenses of 8-week-old *Epha2*$^{+/+}$ (n = 3), *Epha2*$^{+/-}$ and *Epha2*$^{-/-}$ (n = 2) mice on C57BL/6J background by RT-PCR using gene-specific primers. *B2m* mRNA was amplified for normalization of expression. PCR products of the expected sizes of 647 bp and 193 bp for *Bip* and *B2m*, respectively, were obtained. Mean normalized *Bip* expression in lenses of each genotype is indicated. M, molecular size of DNA markers in base pairs; RT^{-}, without reverse transcriptase; PCR^{-}, PCR without template; SD, standard deviation.

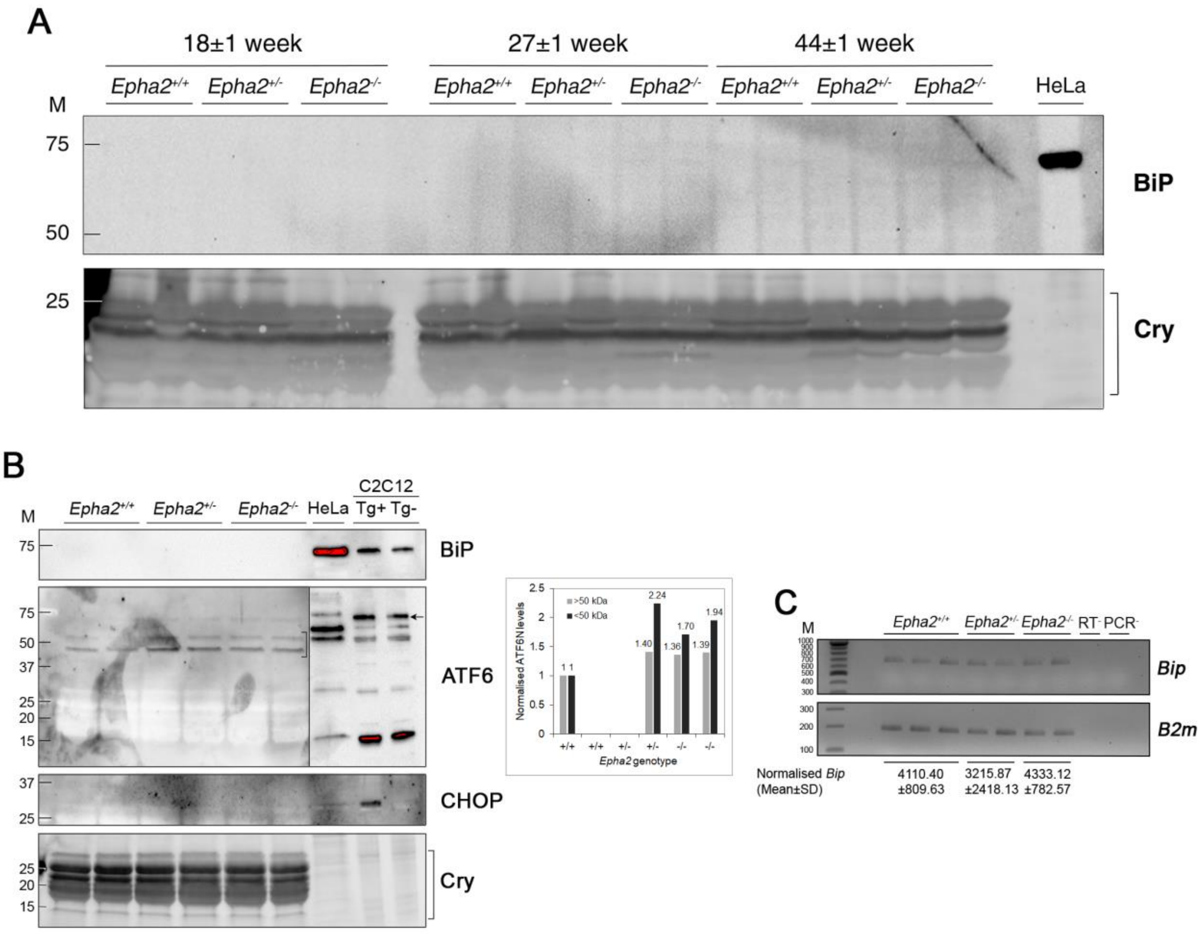

Supplementary Figure S5: Expression of mutant EPHA2- β-galactosidase fusion protein in *Epha2*-knockout mouse lens.

A. Total soluble proteins extracted from lenses (n=2) of 18±1 week-old *Epha2*$^{+/+}$, *Epha2*$^{+/-}$ and *Epha2*$^{-/-}$ mice on C57BL/6J background were analysed by western blotting using anti-β-galactosidase antibody. HeLa cell extract was used as negative control. The partial EPHA2-β-galactosidase fusion protein was detected in *Epha2*$^{+/-}$ and *Epha2*$^{-/-}$ lenses (arrowhead, top panel); the protein migrated slower than its expected size of 163 kDa. Note greater protein intensity in *Epha2*$^{-/-}$ compared to *Epha2*$^{+/-}$ lenses. As expected, a similar sized band was undetectable in *Epha2*$^{+/+}$ lenses and HeLa cell lysate (top panel). The >250 kDa band seen in all the lanes is non-specific. Protein bands corresponding to lens crystallins were visible in all lens lysates and served as internal loading control (bottom panel). **B.** Total insoluble proteins extracted from insoluble pellets following extraction of soluble proteins from lenses (n = 2) of 43±1 week-old *Epha2*$^{+/+}$ and *Epha2*$^{+/-}$ and 19±1 week-old *Epha2*$^{-/-}$ mice on C57BL/6J background were analysed as described above. Total soluble proteins from a *Epha2*$^{-/-}$ lens were used as positive control. The partial EPHA2-β-galactosidase fusion protein was detected in the positive control (arrowhead, top panel) but not in any of the insoluble protein extracts. The >250 kDa band seen in the positive control is non-specific; red indicates signal saturation. Protein bands corresponding to lens crystallins were visible in all insoluble protein extracts and served as loading controls (bottom panel). M, molecular weight of protein standards in kiloDaltons.

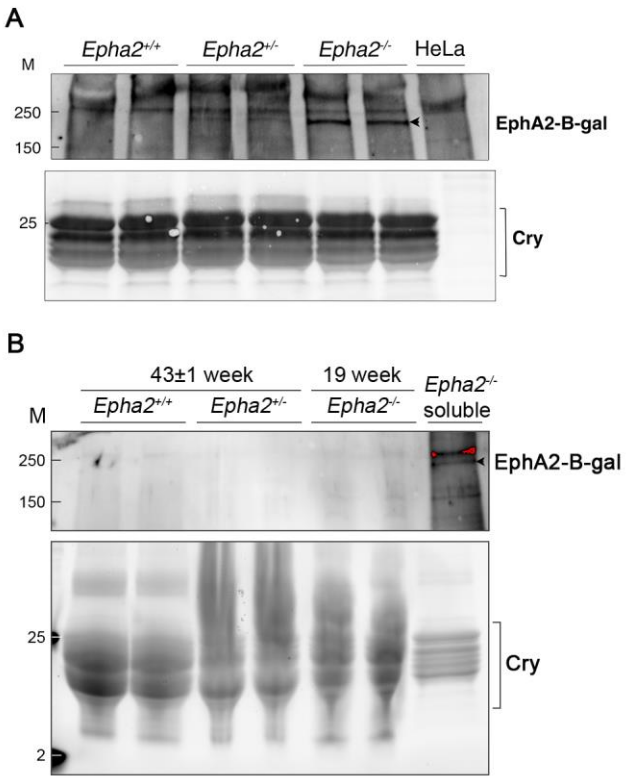
A
M
Epha2+/+
Epha2+/-
Epha2-/-
HeLa
250
150
EphA2-B-gal
25
Cry
B
43±1 week
19 week
Epha2+/+
Epha2+/-
Epha2-/-
Epha2-/-
soluble
M
250
150
EphA2-B-gal
25
Cry
2

Supplementary Figure S6: Comparison of glutathione levels in *Epha2*$^{+/+}$ and *Epha2*$^{-/-}$ mouse lenses.

Concentrations of total soluble GSH (A), GSH in its free, reduced form (B), and oxidized forms as soluble GSSG (C) and protein-bound PSSG (D) were compared between 22-week-old *Epha2*$^{-/-}$ and age-matched *Epha2*$^{+/+}$ mouse lenses (n=3) as described in Materials and Methods. Mean concentrations of total GSH, free GSH, and GSSG expressed as nmol/ mg of lens protein and PSSG as nmol/mg dried tissue have been plotted. Two-tailed t-test p value of each comparison has been indicated. Error bars represent standard deviation from the mean.

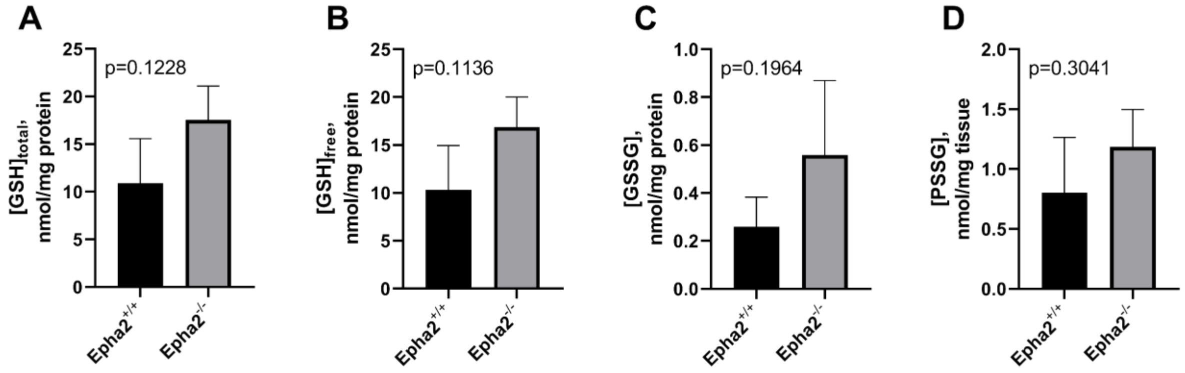

Supplementary Figure S7: Effect of homozygous *Bfsp2* mutation on anterior cortical cataract progression in *Epha2*$^{-/-}$ mice on FVB:C57BL/6J mixed background.

Epha2$^{+/+}$ and *Epha2*$^{-/-}$ mice on FVB:C57BL/6J (50:50) mixed background with *Bfsp2*$^{-/-}$ or *Bfsp2*$^{+/+}$ genotype were monitored for cataract development from 11±3 to 64±3 weeks of age. Mean cortical cataract grade in each genotype of mice at each time-point is graphically represented. No significant difference in ACC severity was observed between *Epha2*$^{-/-}$ mice with *Bfsp2*$^{-/-}$ or *Bfsp2*$^{+/+}$ genotype at any time-point (blue traces); both types of mice developed severe cataract by 38±3 weeks of age. *Epha2*$^{+/+}$ mice with and without the *Bfsp2* mutation (green traces) exhibited

similar mild cataract up to 64±3 weeks of age. Data were compared by Mann Whitney *U* test. Error bars represent standard error of the mean. After an animal exhibited a cataract grade of 4 or 5, the same grade was used for subsequent time-points for data analysis assuming no further increase in cataract severity to occur.

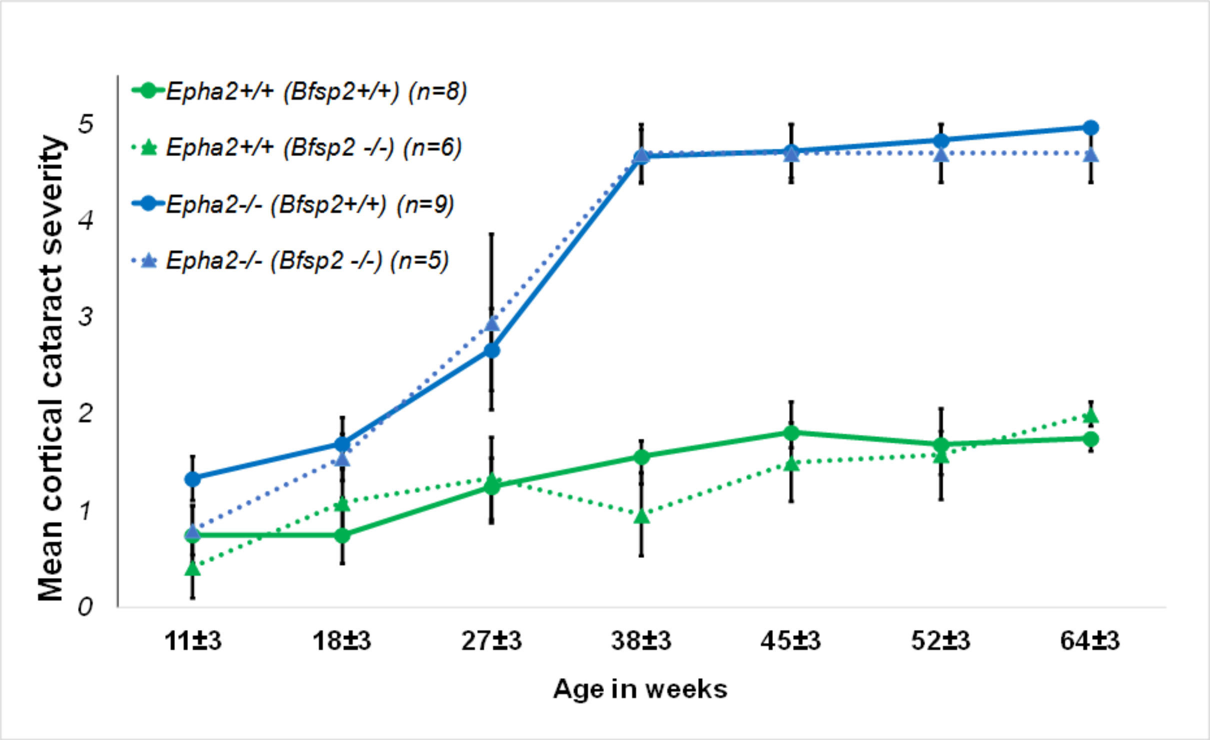

Supplementary Figure 7

Supplementary Figure S8: The effect of heterozygous *Bfsp2* mutation on anterior cortical cataract progression in *Epha2*$^{-/-}$ and *Epha2*$^{+/-}$ mice on FVB:C57BL/6J mixed background.

Epha2$^{+/+}$, Epha2$^{+/-}$ and *Epha2*$^{-/-}$ mice on FVB:C57BL/6J (50:50) mixed background with *Bfsp2*$^{+/-}$ or *Bfsp2*$^{+/+}$ genotype were monitored for cataract development from 11±3 to 64±3 weeks of age. Mean cortical cataract grade in each genotype of mice at each time-point is graphically represented. No significant difference in ACC severity was observed between *Epha2*$^{-/-}$ mice with *Bfsp2*$^{+/-}$ or *Bfsp2*$^{+/+}$ genotype (blue traces), and between *Epha2*$^{+/-}$ mice with *Bfsp2*$^{+/-}$ or *Bfsp2*$^{+/+}$ genotype (red traces) at any time-point. Both types of *Epha2*$^{-/-}$ mice developed severe cataract by 38±3 weeks of age. Both types of *Epha2*$^{+/-}$ mice developed only moderate cataract up to 64±3 weeks of age. *Epha2*$^{+/+}$ mice with and without the *Bfsp2* mutation exhibited similar mild cataract up to 64±3 weeks of age (green traces); the mild cataract was significantly different at 18±3 weeks of age (p=0.014) but the difference did not persist at the subsequent time-points. Data were compared by Mann Whitney U test. Error bars represent standard error of the mean. After an animal exhibited a cataract grade of 4 or 5, the same grade was used for subsequent time-points for data analysis assuming no further increase in cataract severity occurred.

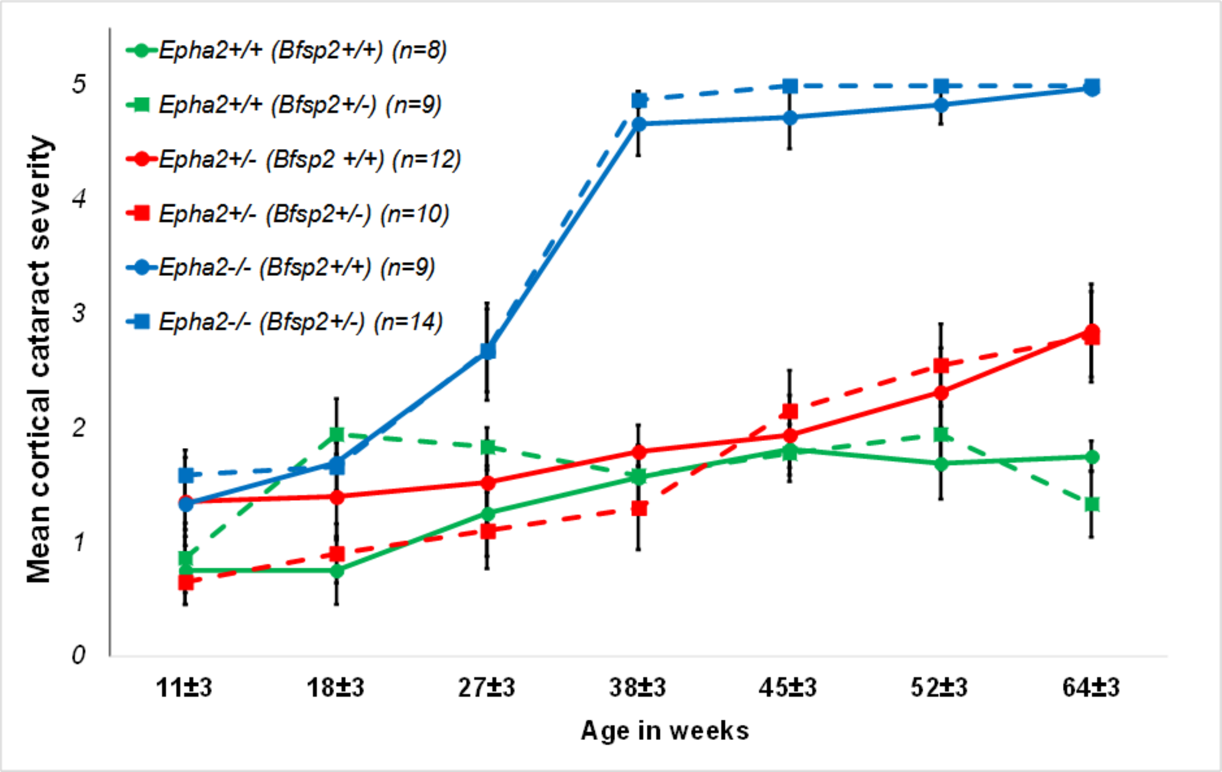

Supplementary Figure 8
